# Candidate genes identification and RNA-seq based pathway analysis associated with primary angle-closure glaucoma with cataract

**DOI:** 10.1186/s12886-023-02950-0

**Published:** 2023-05-02

**Authors:** Min Liu, Fei Hu, Caifeng Lei, Min Fu, Xue Li, Ling Yu

**Affiliations:** 1 Department of Ophthalmology, The People’s Hospital of Wenjiang Chengdu, Chengdu, Sichuan Province 611130 China; 2 Department of Ophthalmology, Daping Hospital, Army Medical Center, Army Medical University, Chongqing, 40042 China

**Keywords:** Age-related cataract, Bioinformatics, Differentially expressed genes, Primary angle-closure glaucoma

## Abstract

**Background:**

Cataract is commonly observed in patients with primary angle-closure glaucoma; however, its underlying pathological mechanisms remain unclear. This study aimed to improve our knowledge on the pathological processes involved in primary angle-closure glaucoma (PACG) by identifying potential prognostic genes associated with cataract progression.

**Methods:**

Thirty anterior capsular membrane samples were collected from PACG patients with cataracts and age-related cataracts. Differentially expressed genes (DEGs) between these two cohorts were analyzed using high-throughput sequencing. Gene ontology and Kyoto Encyclopedia of Genes and Genomes analyses were performed to screen the DEGs, and potential prognostic markers and their coexpression network were then predicted by bioinformatic analyses. The DEGs were further validated by reverse transcription-quantitative polymerase chain reaction.

**Results:**

A total of 399 DEGs were found to be specifically associated with cataracts development in PACG patients, among which 177 and 221 DEGs were upregulated and downregulated, respectively. STRING and Cytoscape network analyses revealed seven genes—*CTGF*, *FOS*, *CAV1*, *CYR61*, *ICAM1*, *EGR1*, and *NR4A1*—that were remarkably enriched and mainly involved in the MAPK, PI3K/Akt, Toll-like receptor, and TNF signaling pathways. RT-qPCR-based validation further confirmed that the sequencing results were accurate and reliable.

**Conclusions:**

Herein, we identified seven genes and their signaling pathways that may contribute to cataract progression in patients with high intraocular pressure. Taken together, our findings highlight new molecular mechanisms that may explain the high incidence of cataracts in PACG patients. In addition, the genes identified herein may represent new foundations for the development of therapeutic strategies for PACG with cataract.

**Supplementary Information:**

The online version contains supplementary material available at 10.1186/s12886-023-02950-0.

## Background

Age-related cataract (ARC) and glaucoma are the leading causes of visual impairment and irreversible blindness worldwide [1, 2]. The number of glaucoma patients is expected to exceed 111.8 million by 2040 [3]. In China, 6.0–14.1% people suffer of binocular blindness due to primary angle-closure glaucoma (PACG), which is a much higher incidence than in other types of glaucoma [4]. With the aging of the population, cataract and glaucoma often coexist, and the occurrence and development of these two eye diseases often affect each other, with the incidence of cataract in glaucoma patients being usually high [5].

As a neurodegenerative disease, the mechanism of cell death in glaucoma is closely related to oxidative stress [6]. As reported by Fan Gaskin et al., reactive oxygen species (ROS) produced by NADPH oxidase are important contributors for oxidative stress in glaucoma [7]. Excessive ROS can lead to apoptosis of human lens epithelial cells (LECs), representing one of the main trigger factors of ARC [8, 9]. By analysis the aqueous humor protein profile in PACG patients with cataracts, Adav et al. [10] identified 1,376 different aqueous humor proteins, among which superoxide dismutase, catalase, and peroxiredoxin-2 were significantly increased in PACG patients, indicating a higher level of oxidative stress as compared with patients with cataracts only. Cheng et al. also revealed that *AQP1* levels were remarkably increased in PACG compared with the ARC group, which can lead to an increase in the lens thickness [11]. Thus, we hypothesized that continuous presence of oxidative stress in PACG patients with long-term high intraocular pressure (IOP) may contribute for abnormal gene expression in lens epithelial cells (LECs). Our study aimed to explore key molecules and pathways involved in PACG with cataract and to elucidate the mechanisms of oxidative damage and apoptosis in human LECs, which may provide new foundations for disease treatment and clinical practice.

## Materials and methods

### Clinical sample collection

Samples of the anterior capsule were extracted from inpatients who visited the Ophthalmology Department of Army Medical Center Daping Hospita from March to August 2021. The study enrolled 15 PACG patients with cataract (hereafter named BQ group) and 15 ARC patients (hereafter indicated as DB group), who did not differed significantly concerning age and gender (Table 1, *P* > 0.05). All patients in the BQ group had two or more quadrants of iridotrabecular contact and the resulting peripheral anterior synechiae and raised IOP, together with optic nerve damage and visual field defects, diagnosed according to the European Glaucoma Society guidelines [12]. Patients with other ocular, systemic diseases as well as history of eye laser therapy and surgery were excluded from the study. Intraoperatively, the central anterior capsule of the lens was obtained using capsulorhexis forceps with a diameter of 5–5.5 mm, and the anterior capsule specimen was obtained using toothless forceps. The blood and surface viscoelastic agents were then washed with sterile saline, and the cleaned samples were placed in EP sterilization tubes. We used the RNA extraction method reported by Wang Z et al. [13]. Briefly, five samples from the same cohort were randomly mixed, placed in ice boxes, and stored at − 80 °C until analysis. The study was conducted in accordance with the Declaration of Helsinki, and all subjects were well informed regarding the study and its potential risks and signed an informed consent form before participation.

**Table 1:** General patient information

Subgroups	PACG with cataract	ARC	*P*
**Age (SD)**	68.13 (8.008)	69.53 (6.468)	0.603
**Gender(M/F)**	6/9	7/8	0.713

Note: There were no significant differences in age and gender between the experimental and control groups (*P* > 0.05). Gender and age were performed using chi-square tests and one-way ANOVA, respectively

### RNA extraction

Trizol was added to the crushed samples, and total exogenous RNA was extracted using a miRNeasy Mini Kit (217,004; Qiagen, Hilden, Germany) and RNase-free DNase Set (79,254; Qiagen). A QubitTM RNA HS Assay Kit (1,875,983; Thermo Fisher Scientific, Waltham, MA, USA) was used for the quantitative and qualitative control of the RNA samples, and the VAHTS Stranded mRNA-seq Library Prep Kit for Illumina V2 (NR612-02; Vazyme Biotech, Nanjing, China) was subsequently used to construct the RNA sequence library.

### Data processing

All RNA samples were sequenced using an Illumina Nova Seq 6000 system (Illumina, San Diego, CA, USA) with technical support from Epibiotek Guangzhou Co. Ltd. Quality control of the high-throughput sequencing data was performed using the *Fastqc* algorithm, connectors and low-quality bases were removed using *Cutadapt*, and then a rapid comparison of RNA-sequencing reads with genomes was performed using *Hisat2* algorithm. Finally, the genomic alignment results of the high-throughput sequencing data were normalized according to the fragments per kilobase of transcript per million reads, transformed into gene expression data, and analyzed.

### Analysis of differentially expressed genes (DEGs)

*DESeq2* algorithm was used to analyze the DEGs between the two sample groups. The screening conditions for DEGs were *P* < 0.05 and |log2FoldChange| > 1 [14]. Only genes satisfying these conditions were considered statistically significant and regarded as DEGs.

### Gene set enrichment analysis

Gene ontology (GO) analysis (http://geneontology.org/) was used to classify the functions of the DEGs from three perspectives: biological process, cellular component, and molecular function. The Kyoto Encyclopedia of Genes and Genomes (KEGG) analysis (http://www.genome.jp/kegg/) was used to predict the possible involvement of these DEGs in signaling pathways [15]. GO and pathway terms with significant enrichment were screened using these analysis methods. Fisher’s test was used to calculate the significance levels (*P*-values) of all GO terms and pathways at a screening *P*-value of < 0.05.

### Protein-protein interaction (PPI) network construction

The Search Tool for the Retrieval of Interacting Genes (STRING database, http://string-db.org/) is an online database that collects predicted and experimental protein interaction data. The DEGs were imported into the STRING database to obtain a PPI network interaction map, which was combined with Cytoscape to screen for target genes using the Cytohubba plug-in. ClueGO and CluePedia tools of Cytoscape were used to visualize the target gene enrichment pathways.

### Quantitative real-time polymerase chain reaction (RT-qPCR) validation

Thirty additional anterior capsule samples collected from PACG with cataract and ARC patients were used for RT-PCR. Five samples from the same group were mixed together to extract total RNA, and the procedure was repeated three times. The expression of the screened genes was determined using SYBRGreen, with 10 µL reaction components containing 1 ng cDNA, 5 µL 2×SG Fast qPCR Master Mix, 2.5 nM forward primer, 2.5 nM reverse primer, 1 µL DNF Buffer, and RNase-free ddH_2_O added to a total volume of 10 µL. The reaction program consisted of 45 cycles at 95 °C for 5 min, followed by incubation at 95 °C for 15 s and 58 °C for 1 min. The 2^−ΔΔCt^ method was used to calculate the fold expression change, using as internal reference *GAPDH*.

### Statistical analysis

The statistical significance of all experimental data was tested using Student’s *t*-tests. The significance level *P* < 0.05 was considered acceptable.

## Results

### Quality control and preprocessing of the data

Three PACG with cataract samples (BQ1, BQ2, and BQ3) and three ARC samples (DB1, DB2, and DB3) were sequenced. To reduce the impact of data errors, *Fastqc* was used to remove from the sequencing data the low-quality sequences below Q20, contaminated sequences, and sequences that were too short. The high-quality filtered data were quickly compared with the reference genome GRCh38.91 using Hisat2. Overall, the total number of reads in BQ1, BQ2, and BQ3 were of 48,877,224; 45,994,552; and 41,789,584, including 85.3%, 85.6%, and 93.3% mapped reads, respectively. The total number of reads in DB1, DB2, and DB3 were of 41,789,584; 42,488,576; and 45,243,504, including 90.4%, 92%, and 86.8% mapped reads, respectively (Table 2).

**Table 2:** Statistics of reference genome alignment results

Sample	Total Reads	Mapped Reads	Mapped Rate	Unique Mapped Reads	Unique Mapped Rate
BQ1	48,877,224	41,697,981	0.853	38,964,955	0.797
BQ2	45,994,532	39,384,363	0.856	36,853,566	0.801
BQ3	41,789,584	38,998,051	0.933	37,038,101	0.886
DB1	48,779,872	44,091,616	0.904	41,242,874	0.845
DB2	42,488,576	39,076,821	0.92	37,016,338	0.871
DB3	45,243,504	39,279,062	0.868	36,724,648	0.812

### Analysis of DEGs

**Fig. 1:**
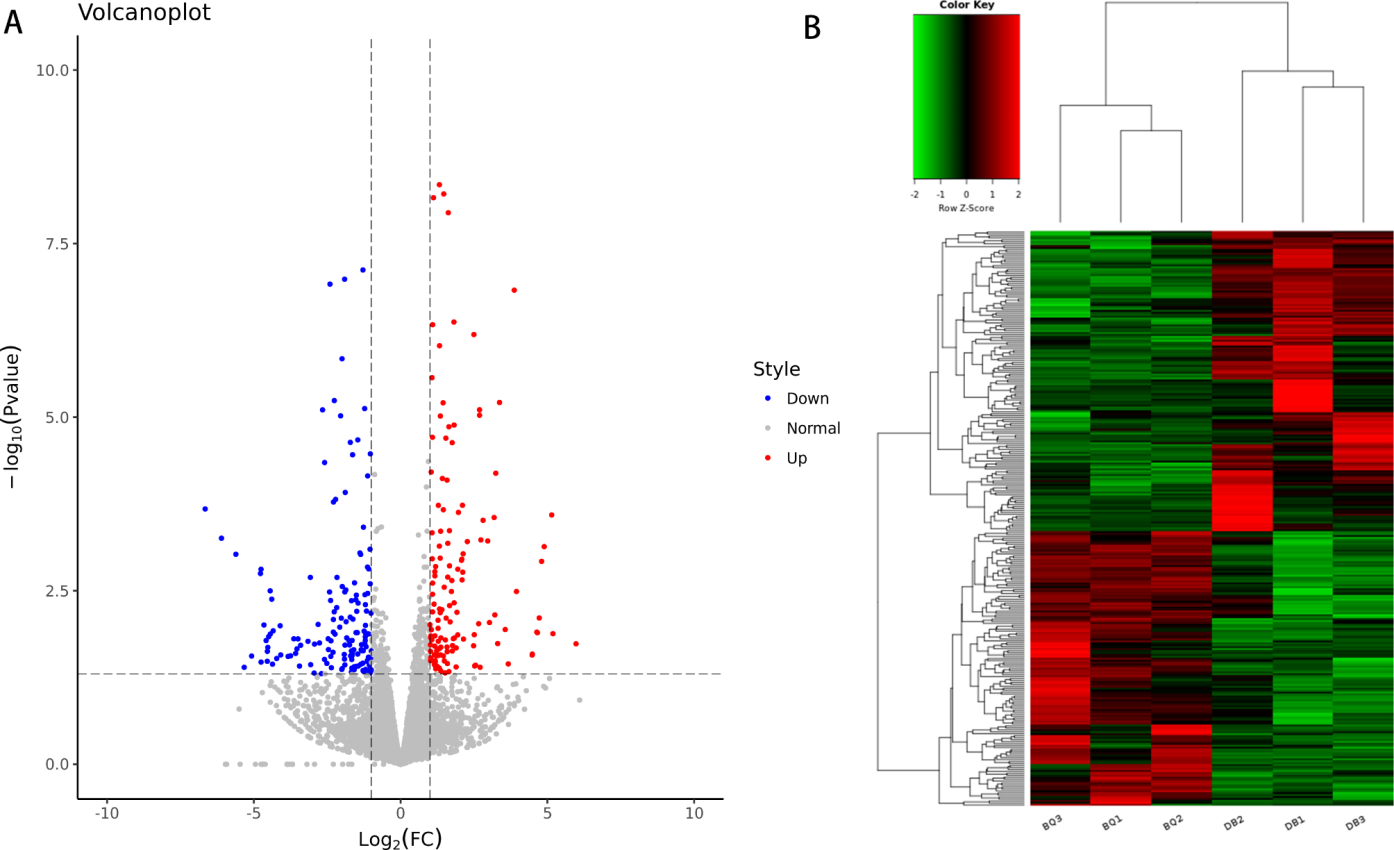
Differentially expressed genes (DEGs) in anterior capsule between the two groups **a** Volcano map of differentially expressed genes; the red and blue dots indicate significantly up- and down-regulated genes, respectively. **b** Heat map of differentially expressed genes; each column and row represent a sample and a specific mRNA, respectively. Red and green represent gene expression higher or lower than the median. The darker the color, the closer it is to the median

Sequencing data analysis revealed 31,233 differentially expressed mRNAs, among which a total of 398 DEGs were screened between the two study groups using the screening criteria of *P* < 0.05 and |log2FoldChange| > 1 (Supplemental Table 1). In 398 DEGs, 291 were protein_coding and 80 were lncRNA. Compared with the DB group, 177 and 221 genes were upregulated and downregulated, respectively, in the BQ group. The candidate genes were then graded and clustered to explore their biological significance, and volcano (Fig. 1A) and cluster (Fig. 1B) plots were used to distinguish their expression patterns (Fig. 1). The volcano map provides a more intuitive picture of the distribution of the different genes in the samples.

### GO enrichment and KEGG pathway analysis

**Fig. 2:**
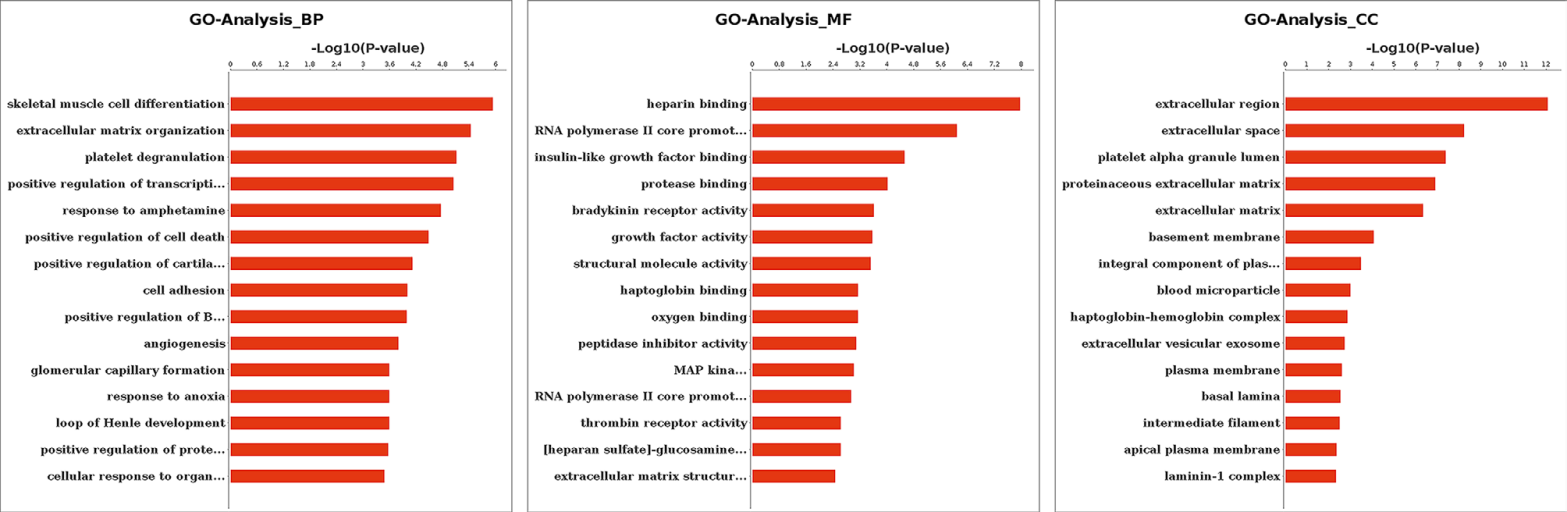
GO enrichment analysis of differential expression genes (DEGs) The figure shows the first 15 GO terms for biological process (BP), molecular function (MF) and cellular component (CC). Y-axis: GO-term; X-axis: log10(*P*-value)

**Fig. 3:**
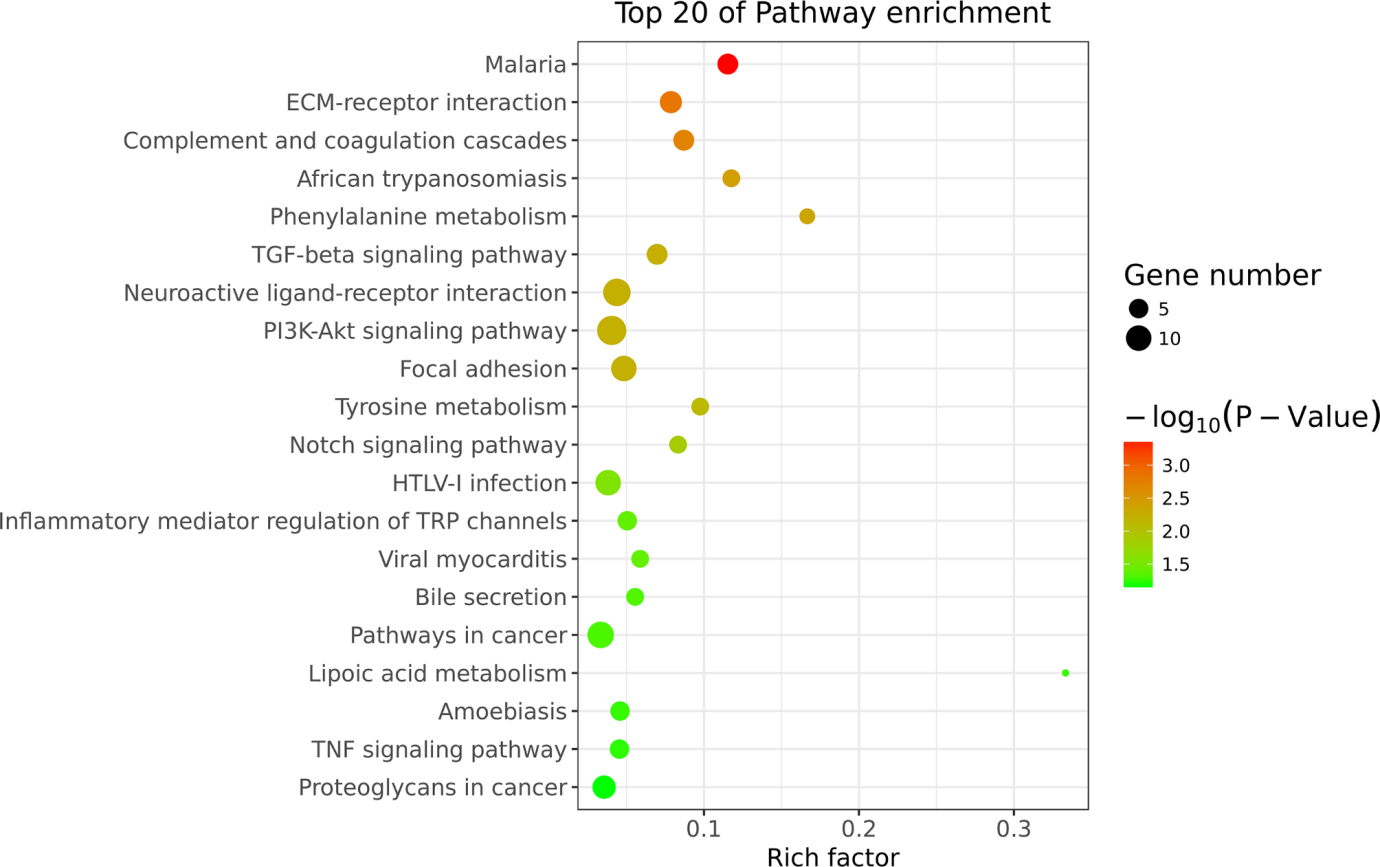
KEGG enrichment analysis of differentially expressed genes (DEGs) Scatter plot of pathway enrichment. The figure shows the top 20 enriched pathways. The Rich factor refers to the ratio of DEGs to the number of annotated genes in the pathway. The larger the Rich factor, the greater the degree of enrichment

To explore the functions of these DEGs, as well as the signaling pathways in which they could be involved, we conducted GO (Fig. 2) and KEGG pathway analyses (Fig. 3). According to the GO analysis results for biological process, the DEGs were significantly enriched in skeletal muscle cell differentiation, extracellular matrix organization, positive regulation of cell death, among other mechanisms. KEGG pathway analysis revealed that the complement and coagulation cascades, focal adhesion, tyrosine metabolism pathway, TGF-β and PI3K/Akt signaling pathway were significantly upregulated in PACG patients with cataract.

### PPI Network analysis

**Fig. 4:**
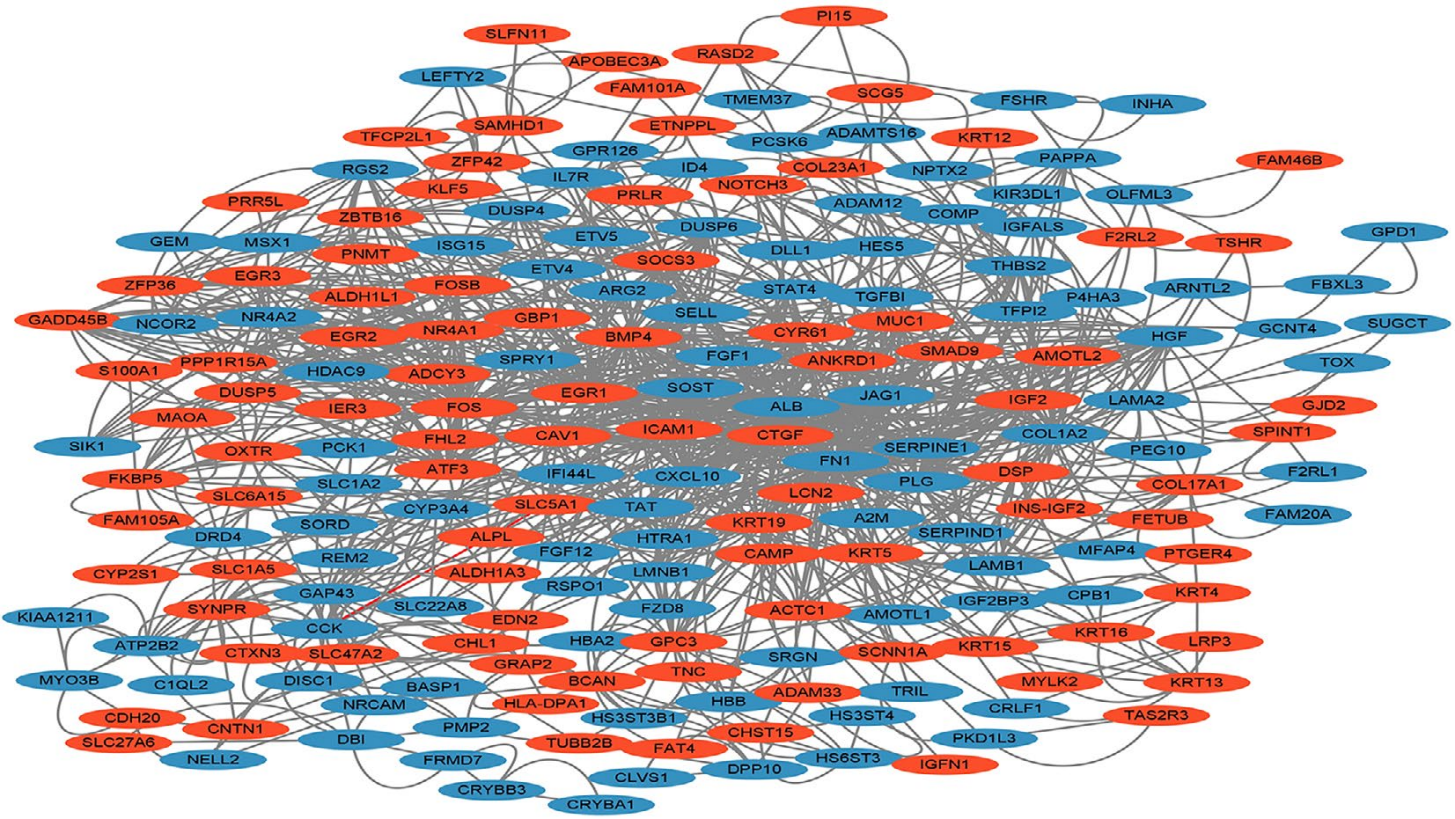
Protein-protein interaction (PPI) network diagram constructed by STRING and Cytoscape Red and blue presents up- and down-regulated genes, respectively

**Fig. 5:**
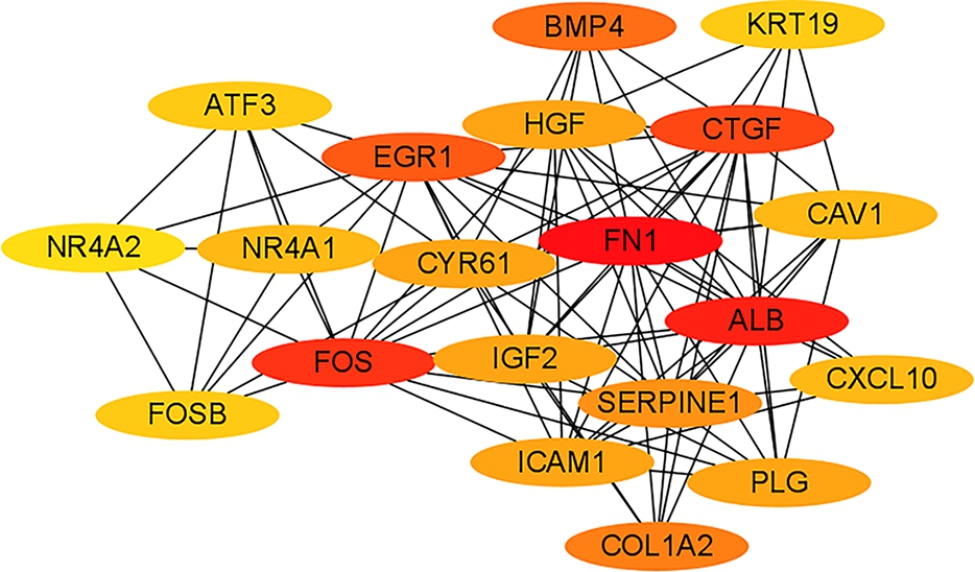
CytoHubba screened 20 target genes The top 20 genes screened out by degree value

The STRING database and Cytoscape platform (www.cytoscape.org) were used to build the PPI networks of the identified DEGs based on information from publicly available databases and text mining, as well as for mining the core genes. The designed PPI network contained 289 nodes and 587 edges, with a comprehensive score > 0.4 (Fig. 4). Next, we used the CytoHubba plug-in to select the 20 genes with the closest connection, according to the degree value (*FN1*, ALB, *FOS*, *CTGF*, *EGR1*, *BMP4, COL1A2, SERPINE1, ICAM1*, *HGF*, *CYR61*, *IGF2, PLG, CXCL10, NR4A1*, *CAV1*, *FOSB, ATF3, KRT19*, and *NR4A2*) (Fig. 5). Based on the expression patterns and pathways involving the above genes, *CTGF* (degree = 52), *FOS* (degree = 70), *CAV1* (degree = 30), *CYR61* (degree = 34), *ICAM1* (degree = 34), *EGR1* (degree = 50), and *NR4A1* (degree = 30) were selected as target genes for the subsequent validation experiments. Noteworthily, the ClueGO and CluePedia tools of Cytoscape also showed that these genes were enriched in the MAPK, PI3K/Akt, Toll-like receptor, TNF, and other important signaling pathways.

### RT-qPCR validation

**Fig. 6:**
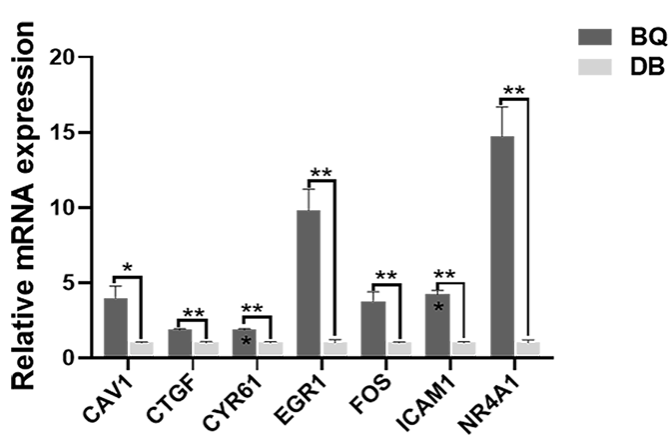
RT-qPCR validation of 7 target genes The relative expression level of genes in PACG with cataract and ARC patients, experiments were repeated three times and are presented as BQ and DB, respectively (*P < 0.05, **P < 0.01, ***P < 0.001)

To verify the above described sequencing results, the seven DEGs (*CTGF*, *FOS*, *CAV1*, *CYR61*, *ICAM1*, *EGR1*, and *NR4A1*) were further evaluated by RT-qPCR. Compared with the ARC group, the levels of *CTGF*, *FOS*, *CAV1*, *CYR61*, *ICAM1*, *EGR1*, and *NR4A1* in the anterior capsule of PACG patients with cataracts were upregulated (Fig. 6), which was consistent with the sequencing data (Supplemental Table 2).

## Discussion

Glaucoma, for which increased IOP is the main risk factor, can cause visual field loss and optic nerve damage, and consequently lead to irreversible blindness [16]. IOP damages the cornea, iris, trabecular meshwork, lens, retina, and other tissues. Clinically, cataract and glaucoma can coexist, which further increases the incidence rate of blindness [17]. Oxidative stress plays a key role in the development of both glaucoma and cataract [18, 19]. Moreover, apoptosis of human LECs, which is most severe under metabolic stress and oxidative damage, is believed to be the main cause of cataract formation [20]. Under the action of oxidative stress, such as due to IOP elevation, ROS production increases, which in turn causes inflammation, excitatory toxicity, vascular injury and hypoxia, glial dysfunction, and changes in axon transport. These events not only cause damage to the retina and trabecular meshwork, but also damage the LECs [21, 22]. To date, metabolomics, proteomics, and transcriptomics studies have identified several molecular changes associated with glaucoma; however, systematic studies of multimolecular interactions and mechanisms are still scarce [23–25]. As an important component of transcriptomics, mRNAs are widely involved in infectious diseases and tumors. By regulating gene expression, cells can adapt rapidly and dynamically to various stimuli. The role of mRNA in glaucoma and cataracts has received significant attention in recent years. Several mRNAs, such as *BLM, Ubc, Ptgs2*, and *Jun*, have been shown to be involved in cataract development; however, the role of differential mRNA expression in cataract progression in glaucoma patients remains unclear [26, 27]. Owing to the instability of mRNA molecules, the study of mRNA is difficult and challenging. Nonetheless, with the recent developments on high-throughput sequencing and bioinformatics technologies, mRNA analysis has become an efficient and easy way to study mRNA-based molecular mechanisms.

In this study, we used bioinformatic technology and transcriptome sequencing to conduct for the first time an in-depth analysis of genes related to PACG complicated by cataracts. Human LECs play the most active role in lens metabolism, being responsible for synthetizing most metabolites. By detecting the differential expression of mRNAs in LECs, we can have a better understanding of the changes in the genetic landscape at different disease states. In our sequencing data, the alignment rate of each sample with the reference genome was higher than 85% and a total of 31,233 genes were identified, among which the expression of 398 genes differed by more than 2-fold between the two study cohorts. Compared with ARC samples, 177 upregulated and 221 downregulated DEGs were identified in PACG patients with cataract. They were found to be associated with various biological processes, such as extracellular matrix organization, positive regulation of transcription from the RNA polymerase II promoter, and positive regulation of the BMP signaling pathway. Noteworthily, some of these processes may be related to the structural components of the lens [28, 29]. KEGG pathway analysis further showed that these genes are involved in extracellular matrix-receptor interactions, and in the TGF-β and PI3K/Akt signaling pathways.

Network analysis further revealed 20 genes that are most probably associated with PACG with cataract. After reviewing the literature and removing the genes with low expression, seven genes (*CTGF*, *FOS*, *CAV1*, *CYR61*, *ICAM1*, *EGR1*, and *NR4A1*) were considered to be crucial for the pathogenesis of cataract with high IOP. PPI analysis indicated that *FOS* may be involved in the MAPK, TNF, and Toll-like receptor signaling pathways, which are closely related to ARC development [30, 31]. According to a study by Li and Spector [32], the rapid upregulation of *c-Fos* in rabbit lens epithelial cells induced by H_2_O_2_ impaired the cell proliferation, differentiation, and viability, thereby affecting the normal lens function. *NR4A1* was predicted to be involved in the MAPK and PI3K/Akt signaling pathways. As previously reported, *NR4A1* has pleiotropic regulatory effects on glucose and lipid metabolism, inflammatory responses, and vascular homeostasis [33]. *NR4A1* can reduce endoplasmic reticulum stress or ROS by enhancing MKP7 expression to reduce pancreatic beta cell apoptosis, which can effectively prevent diabetes [34]. *EGR1* expression is closely related to cell proliferation and regulates the levels of various proliferation-related genes [35]. Qin et al. showed that the TGF-β1/SMAD signaling pathway upregulates EGR1 expression, which in turn promotes NOX4-derived ROS production and scar fibrosis [36]. *CTGF* is regulated by TGF-β2, which causes transdifferentiation and fibrosis of human LECs by participating in extracellular matrix remodeling [37]. *CYR61*, similar to *CTGF*, is a member of the CCN protein family. It plays an important role in physiological processes including embryo development, tissue damage repair, senescence, and angiogenesis [38]. The mRNA levels of CYR61 and CTGF were significantly upregulated under high ROS, which is associated with alveolar fibrosis in mice [39]. Increased expression of *CAV1* is closely related to vesicle transport and cell cycle regulation, and affects the function of LECs, which may lead to the occurrence of cataracts [40]. Zhao et al. [26] reported *Hsf4* knockout mice have high expression of *Fos*, *Egr1*, *Ptgs2*, and *Cyr61*, which may induce increased proliferation of LECs and abnormal differentiation of lens fibrocytes, contributing to cataract development. Additionally, Zhao et al. found that *Cav1* interacts with *Ptgs2* and aids in DNA repair in the lens. *ICAM1*, a cell adhesion molecule, is highly expressed in vascular endothelial, epithelial, and immune cells in response to inflammation and tumor cell stimuli [41]. Fan et al. reported that *ICAM1* is involved in the attachment and growth of LECs to collagen and laminin in vitro, which may play a crucial role in the progression of cataract in patients with type 2 diabetes mellitus [42]. CAV1 and ICAM1 upregulation under oxidative stress is also involved in tumor proliferation and migration, as well as chronic lung inflammation [43, 44]. All the seven identified DEGs were further verified by RT-qPCR and the data were consistent with the high-throughput sequencing results, thus providing more information on the cause of cataract in glaucoma patients at the molecular level.

Small study cohort sizes was a serious limitation of the present study. Additionally, whether the abnormal expression of these target genes involves the above-mentioned pathways or how it affects the phenotype of human LECs warrant further explored. The pathways and mechanisms by which these genes promote cataract development in glaucoma patients will need to be further investigated in the future.

## Conclusion

In summary, we analyzed the genetic landscape of glaucoma patients with cataract and ARC using bioinformatic methods, and the results showed that *CTGF*, *FOS*, *CAV1*, *CYR61*, *ICAM1*, *EGR1*, and *NR4A1* may be involved in cataract progression under high IOP; however, further studies are needed to fully understand their underlying molecular mechanisms.

## Electronic supplementary material

Below is the link to the electronic supplementary material.

Supplementary Material 1

Supplementary Material 2

## Data Availability

The datasets used and/or analysed during the current study are available from the corresponding author on reasonable request.

## List of Abbreviations

ARC: Age-related cataract
DEG: Differentially expressed gene
GO: Gene ontology
IOP: Intraocular pressure
KEGG: Kyoto Encyclopedia of Genes and Genomes
LEC: Lens epithelial cell
PACG: Primary angle-closure glaucoma
PPI: Protein-protein interaction
ROS: Reactive oxygen species
RT-qPCR: Reverse transcription-quantitative polymerase chain reaction
