# Supplementary material for: Candidate genes identification and RNA-seq based pathway analysis associated with primary angle-closure glaucoma with cataract
Source: BMC Ophthalmol. 2023 May 2;23:192. doi: 10.1186/s12886-023-02950-0 (PMC10152770; doi:10.1186/s12886-023-02950-0)
Supplement: Supplementary file 1 — Supplementary Material 1 [file 12886_2023_2950_MOESM1_ESM.docx]

**Supplemental Table 2.** **RT-qPCR results**

RT-qPCR results of seven target genes

| Target | Sample | Control | Expression | Expression SEM | Corrected Expression SEM | Mean Cq | Cq SEM | P-Value |
| --- | --- | --- | --- | --- | --- | --- | --- | --- |
| CAV1 | BQ |  | 3.98739 | 0.78403 | 0.78403 | 29.30 | 0.27753 | 0.017241 |
| CAV1 | DB | C | 1.00000 | 0.06441 | 0.06441 | 28.44 | 0.03080 |  |
| CTGF | BQ |  | 1.85016 | 0.11635 | 0.11635 | 27.37 | 0.06917 | 0.001326 |
| CTGF | DB | C | 1.00000 | 0.08374 | 0.08374 | 25.39 | 0.08313 |  |
| CYR61 | BQ |  | 1.86485 | 0.07829 | 0.07829 | 28.48 | 0.01486 | 0.000030 |
| CYR61 | DB | C | 1.00000 | 0.07104 | 0.07104 | 26.52 | 0.05309 |  |
| EGR1 | BQ |  | 9.81430 | 1.41901 | 1.41901 | 30.08 | 0.20016 | 0.003775 |
| EGR1 | DB | C | 1.00000 | 0.21192 | 0.21192 | 30.51 | 0.29290 |  |
| FOS | BQ |  | 3.77102 | 0.62249 | 0.62249 | 29.98 | 0.23080 | 0.008839 |
| FOS | DB | C | 1.00000 | 0.06626 | 0.06626 | 29.03 | 0.03813 |  |
| ICAM1 | BQ |  | 4.22714 | 0.25515 | 0.25515 | 29.41 | 0.06431 | 0.000070 |
| ICAM1 | DB | C | 1.00000 | 0.06877 | 0.06877 | 28.63 | 0.04645 |  |
| NR4A1 | BQ |  | 14.74670 | 1.93000 | 1.93000 | 29.48 | 0.17945 | 0.001773 |
| NR4A1 | DB | C | 1.00000 | 0.18921 | 0.18921 | 30.50 | 0.25852 |  |
