# Supplementary material for: Candidate genes identification and RNA-seq based pathway analysis associated with primary angle-closure glaucoma with cataract
Source: BMC Ophthalmol. 2023 May 2;23:192. doi: 10.1186/s12886-023-02950-0 (PMC10152770; doi:10.1186/s12886-023-02950-0)
Supplement: Supplementary file 2 — Supplementary Material 2 [file 12886_2023_2950_MOESM2_ESM.docx]

**Supplemental Table 1.** **DEGs statistics results**

The 398 differentially expressed genes (DEGs) in two groups

| ensembl_gene_id | log2FC | Pvalue | FDR | Style | external_gene  _name | gene_biotype | BQ1 | BQ2 | BQ3 | DB1 | DB2 | DB3 |
| --- | --- | --- | --- | --- | --- | --- | --- | --- | --- | --- | --- | --- |
| ENSG00000168398 | 1.643912 | 1.38E-05 | 0.009056 | up | BDKRB2 | protein_coding | 1.53811 | 3.410779 | 4.119746 | 0.790421 | 1.324032 | 0.626579 |
| ENSG00000134986 | -1.04375 | 0.0008 | 0.201046 | down | NREP | protein_coding | 19.29548 | 19.99505 | 15.07296 | 38.53706 | 32.20424 | 34.93465 |
| ENSG00000134940 | -4.10296 | 0.010118 | 1 | down | ACRV1 | protein_coding | 0.027512 | 0 | 0 | 0.203829 | 0.149533 | 0.144697 |
| ENSG00000168309 | 1.119337 | 6.91E-09 | 2.20E-05 | up | FAM107A | protein_coding | 192.0746 | 226.8608 | 242.9931 | 86.98591 | 122.7988 | 80.32686 |
| ENSG00000242880 | -4.15773 | 0.042099 | 1 | down | AC046136.1 | lincRNA | 0 | 0 | 0 | 0.211477 | 0.24823 | 0.600506 |
| ENSG00000168481 | 2.271133 | 0.000621 | 0.16701 | up | LGI3 | protein_coding | 0.357986 | 0.276472 | 0.424324 | 0.102007 | 0.074834 | 0.028966 |
| ENSG00000266993 | 2.886751 | 0.03853 | 0.999963 | up | AL050343.1 | antisense_RNA | 0.235882 | 0.099715 | 0.830792 | 0.087378 | 0.051282 | 0 |
| ENSG00000120693 | 1.086987 | 0.006396 | 0.758658 | up | SMAD9 | protein_coding | 2.742509 | 1.874739 | 5.126706 | 0.917139 | 1.975607 | 1.362242 |
| ENSG00000120738 | 5.145562 | 0.000256 | 0.088974 | up | EGR1 | protein_coding | 13.88593 | 13.5478 | 3.522028 | 0.01796 | 0.927579 | 0.061199 |
| ENSG00000120708 | -1.21735 | 0.003616 | 0.561522 | down | TGFBI | protein_coding | 0.221965 | 0.248378 | 0.431126 | 0.544119 | 0.780611 | 0.618028 |
| ENSG00000252498 | 4.492417 | 0.014272 | 1 | up | RNU6-1016P | snRNA | 1.942264 | 2.736859 | 4.275483 | 0 | 0 | 0 |
| ENSG00000168685 | -4.54339 | 0.032909 | 1 | down | IL7R | protein_coding | 0 | 0 | 0 | 0.012183 | 0.0143 | 0.124541 |
| ENSG00000129195 | -1.59758 | 0.022415 | 0.999963 | down | PIMREG | protein_coding | 0.249929 | 0.429215 | 0.171927 | 1.359783 | 0.543353 | 0.525782 |
| ENSG00000120875 | -2.40628 | 1.21E-07 | 0.000211 | down | DUSP4 | protein_coding | 2.055331 | 3.868032 | 2.225897 | 22.03658 | 10.44211 | 8.289324 |
| ENSG00000203722 | -1.525 | 0.004277 | 0.623329 | down | RAET1G | protein_coding | 1.979109 | 2.730679 | 1.089148 | 7.942144 | 3.585543 | 4.279161 |
| ENSG00000144893 | -2.23531 | 0.013355 | 0.999963 | down | MED12L | protein_coding | 0.03965 | 0.035917 | 0.037407 | 0.298999 | 0.215503 | 0.011916 |
| ENSG00000105281 | 1.79445 | 0.015539 | 0.999963 | up | SLC1A5 | protein_coding | 0.992473 | 1.138142 | 0.836701 | 0.215109 | 0.665664 | 0.022212 |
| ENSG00000144908 | 1.514518 | 0.048364 | 0.999963 | up | ALDH1L1 | protein_coding | 0.370116 | 0.293362 | 0.581951 | 0.012241 | 0.273004 | 0.13904 |
| ENSG00000237061 | 2.025886 | 0.017252 | 0.999963 | up | AC100823.1 | sense_intronic | 1.9018 | 1.607905 | 2.511847 | 0.117414 | 1.102554 | 0.266725 |
| ENSG00000071575 | -1.28415 | 7.59E-08 | 0.000161 | down | TRIB2 | protein_coding | 4.407359 | 4.62854 | 5.552643 | 11.01247 | 15.36696 | 7.799465 |
| ENSG00000262179 | -1.67767 | 0.046967 | 0.999963 | down | MYMX | protein_coding | 0.297956 | 1.495726 | 0.491916 | 2.000491 | 0.728739 | 3.682564 |
| ENSG00000237352 | -4.42543 | 0.028232 | 0.999963 | down | LINC01358 | lincRNA | 0.023634 | 0 | 0 | 0.481511 | 0.051381 | 0 |
| ENSG00000237481 | 2.34953 | 0.041794 | 0.999963 | up | AL117350.1 | antisense_RNA | 0.76072 | 0.401976 | 0.941942 | 0.176121 | 0.103364 | 0.100022 |
| ENSG00000081181 | -1.00026 | 0.023358 | 0.999963 | down | ARG2 | protein_coding | 1.815312 | 2.075731 | 1.179155 | 4.244125 | 2.490857 | 2.848542 |
| ENSG00000105664 | -6.1023 | 0.000555 | 0.155911 | down | COMP | protein_coding | 0 | 0 | 0 | 0.041639 | 0.855325 | 0.070943 |
| ENSG00000237575 | -2.7738 | 0.020565 | 0.999963 | down | PYY2 | Unprocessed  _pseudogene | 0.056559 | 0.239094 | 0 | 0.576157 | 0.245923 | 1.011372 |
| ENSG00000115112 | 1.731223 | 0.003251 | 0.524845 | up | TFCP2L1 | protein_coding | 2.521542 | 1.578142 | 3.863825 | 0.169828 | 1.452353 | 0.702693 |
| ENSG00000164038 | 1.151736 | 0.026538 | 0.999963 | up | SLC9B2 | protein_coding | 0.661919 | 0.555514 | 0.79282 | 0.216348 | 0.507895 | 0.163823 |
| ENSG00000164047 | 2.073085 | 0.001153 | 0.259097 | up | CAMP | protein_coding | 50.48321 | 171.625 | 44.23547 | 32.68047 | 8.524448 | 17.3568 |
| ENSG00000164056 | -1.20344 | 0.016562 | 0.999963 | down | SPRY1 | protein_coding | 0.23335 | 0.369917 | 0.359569 | 0.756347 | 0.659503 | 0.638176 |
| ENSG00000164089 | 3.665913 | 0.035874 | 1 | up | ETNPPL | protein_coding | 0.057987 | 0.245131 | 0.127647 | 0 | 0.031517 | 0 |
| ENSG00000071909 | -1.99304 | 0.042017 | 0.999963 | down | MYO3B | protein_coding | 0 | 0.023265 | 0.109033 | 0.183479 | 0.155542 | 0.115778 |
| ENSG00000213659 | 4.181476 | 0.041603 | 1 | up | RSU1P3 | Processed_  pseudogene | 0.291533 | 0.385127 | 0.080219 | 0 | 0 | 0 |
| ENSG00000130881 | 1.890769 | 0.021121 | 0.999963 | up | LRP3 | protein_coding | 0.367671 | 0.304095 | 0.281513 | 0.118432 | 0.139014 | 0 |
| ENSG00000188112 | 1.06299 | 0.016091 | 0.999963 | up | C6orf132 | protein_coding | 0.558596 | 0.486773 | 0.51774 | 0.154283 | 0.383497 | 0.195856 |
| ENSG00000105825 | -6.6575 | 0.00021 | 0.07872 | down | TFPI2 | protein_coding | 0 | 0 | 0 | 0.09224 | 1.488719 | 0.026192 |
| ENSG00000140263 | -1.11474 | 0.045335 | 0.999963 | down | SORD | protein_coding | 1.363686 | 1.270864 | 1.105233 | 4.982616 | 1.536254 | 1.13449 |
| ENSG00000154898 | 1.803459 | 0.046436 | 0.999963 | up | CCDC144CP | Processed_  pseudogene | 0.093143 | 0.147655 | 0.24348 | 0.010782 | 0.101249 | 0.024494 |
| ENSG00000164220 | 1.014017 | 0.019042 | 0.999963 | up | F2RL2 | protein_coding | 0.709916 | 0.637723 | 0.937639 | 0.328718 | 0.347261 | 0.373368 |
| ENSG00000178882 | 2.128649 | 0.000931 | 0.226922 | up | RFLNA | protein_coding | 0.688954 | 0.88991 | 0.533614 | 0.212674 | 0.138686 | 0.107361 |
| ENSG00000164251 | -4.44363 | 0.003178 | 0.523215 | down | F2RL1 | protein_coding | 0 | 0 | 0.091946 | 0.077363 | 1.861554 | 0.043935 |
| ENSG00000139318 | -1.71642 | 0.007626 | 0.832853 | down | DUSP6 | protein_coding | 0.53942 | 0.467754 | 0.365359 | 1.06313 | 3.382838 | 0.349167 |
| ENSG00000188269 | 2.966617 | 0.000608 | 0.165771 | up | OR7A5 | protein_coding | 0.360873 | 0.25934 | 0.683171 | 0.026736 | 0.141219 | 0 |
| ENSG00000129965 | 3.562769 | 0.011467 | 0.995415 | up | INS-IGF2 | protein_coding | 0.106357 | 0.224803 | 0.257535 | 0 | 0 | 0.044749 |
| ENSG00000105974 | 1.041805 | 6.17E-05 | 0.030229 | up | CAV1 | protein_coding | 20.51401 | 16.25486 | 20.77904 | 10.21182 | 9.506562 | 6.799355 |
| ENSG00000105967 | 2.707606 | 0.040127 | 0.999963 | up | TFEC | protein_coding | 0.036728 | 0.048519 | 0.111166 | 0.017006 | 0.009981 | 0 |
| ENSG00000213892 | -4.75433 | 0.001554 | 0.312412 | down | CEACAM16 | protein_coding | 0 | 0 | 0.039588 | 0.499634 | 0.351879 | 0.189166 |
| ENSG00000188404 | -2.46943 | 0.015607 | 0.999963 | down | SELL | protein_coding | 0.049965 | 0.079207 | 0.027497 | 0.208222 | 0.162939 | 0.420453 |
| ENSG00000140479 | -1.22554 | 7.53E-06 | 0.006258 | down | PCSK6 | protein_coding | 1.628758 | 1.73591 | 1.89903 | 4.20549 | 5.026388 | 2.598887 |
| ENSG00000115468 | 1.179177 | 0.034048 | 0.999963 | up | EFHD1 | protein_coding | 0.646561 | 0.597893 | 0.741286 | 0.17464 | 0.380696 | 0.283373 |
| ENSG00000115414 | -4.77551 | 0.001788 | 0.348525 | down | FN1 | protein_coding | 0.07963 | 0.063117 | 0.014607 | 0.024581 | 3.700428 | 1.012106 |
| ENSG00000272324 | -1.43774 | 0.015521 | 0.999963 | down | AC012629.2 | antisense_RNA | 8.717002 | 8.1888 | 3.908797 | 21.37743 | 7.545333 | 22.92276 |
| ENSG00000272338 | -1.03653 | 0.021402 | 0.999963 | down | AC067838.1 | lincRNA | 2.953228 | 3.566929 | 3.250454 | 6.055871 | 4.586015 | 7.655045 |
| ENSG00000164616 | -1.80873 | 0.016311 | 0.999963 | down | FBXL21 | unitary_  pseudogene | 0.248583 | 0.047765 | 0.124364 | 0.627835 | 0.368473 | 0.404098 |
| ENSG00000091136 | -1.4597 | 2.13E-05 | 0.012704 | down | LAMB1 | protein_coding | 2.38533 | 3.480169 | 3.415345 | 5.639777 | 13.8479 | 5.573186 |
| ENSG00000091129 | -4.39021 | 0.004192 | 0.620572 | down | NRCAM | protein_coding | 0.063725 | 0.028863 | 0.060119 | 2.284697 | 0.791662 | 0.009576 |
| ENSG00000188536 | -2.58937 | 4.51E-05 | 0.022658 | down | HBA2 | protein_coding | 5.610309 | 4.22075 | 1.779225 | 12.6807 | 13.43738 | 38.20831 |
| ENSG00000115641 | 2.808067 | 0.000306 | 0.100804 | up | FHL2 | protein_coding | 0.224429 | 0.171299 | 0.576371 | 0.046186 | 0.054213 | 0.02623 |
| ENSG00000164692 | -2.30277 | 0.024396 | 0.999963 | down | COL1A2 | protein_coding | 0.02031 | 0.075123 | 0.011177 | 0.04702 | 0.397383 | 0.106814 |
| ENSG00000163630 | 1.549153 | 0.007861 | 0.834906 | up | SYNPR | protein_coding | 0.921064 | 0.735992 | 0.642874 | 0.14563 | 0.244198 | 0.354451 |
| ENSG00000163631 | -1.2817 | 0.044822 | 0.999963 | down | ALB | protein_coding | 2.151395 | 0.994727 | 1.154362 | 1.867831 | 1.666256 | 5.85545 |
| ENSG00000187608 | -1.00714 | 0.006669 | 0.761718 | down | ISG15 | protein_coding | 7.568584 | 5.958226 | 7.055271 | 16.52139 | 9.9062 | 11.94168 |
| ENSG00000100053 | -1.88647 | 0.000122 | 0.05045 | down | CRYBB3 | protein_coding | 26.32249 | 24.03512 | 18.07834 | 115.9026 | 35.86163 | 80.70037 |
| ENSG00000173110 | -4.54086 | 0.023672 | 0.999963 | down | HSPA6 | protein_coding | 0.025667 | 0 | 0 | 0.57048 | 0.055802 | 0 |
| ENSG00000148123 | -1.12236 | 0.003464 | 0.546661 | down | PLPPR1 | protein_coding | 8.512745 | 10.33699 | 7.436542 | 24.8701 | 12.80642 | 15.70201 |
| ENSG00000100170 | 2.550299 | 0.037459 | 0.999963 | up | SLC5A1 | protein_coding | 0.192397 | 0.050833 | 0.05294 | 0.011136 | 0.013071 | 0.025297 |
| ENSG00000089327 | 1.365759 | 0.026661 | 0.999963 | up | FXYD5 | protein_coding | 1.155524 | 2.360974 | 0.890275 | 0.32103 | 0.628037 | 0.688757 |
| ENSG00000089356 | 4.720559 | 0.007812 | 1 | up | FXYD3 | protein_coding | 0.124453 | 0.306894 | 0.228298 | 0 | 0 | 0 |
| ENSG00000090339 | 1.046063 | 0.011546 | 0.997764 | up | ICAM1 | protein_coding | 3.836349 | 9.275614 | 12.21423 | 4.349947 | 5.004214 | 2.125924 |
| ENSG00000163884 | 1.026127 | 0.029357 | 0.999963 | up | KLF15 | protein_coding | 5.343363 | 3.398214 | 7.676713 | 1.357513 | 5.011611 | 1.591644 |
| ENSG00000124253 | -2.51398 | 0.035281 | 0.999963 | down | PCK1 | protein_coding | 0.035982 | 0.050703 | 0 | 0.344328 | 0.039113 | 0.088312 |
| ENSG00000100276 | 1.59407 | 0.035467 | 0.999963 | up | RASL10A | protein_coding | 1.415293 | 0.71047 | 0.506264 | 0.327667 | 0.499996 | 0.074435 |
| ENSG00000100359 | -4.22294 | 0.029973 | 1 | down | SGSM3 | protein_coding | 0 | 0 | 0 | 0.053446 | 0.047051 | 0.045529 |
| ENSG00000100351 | 2.688306 | 7.84E-06 | 0.006258 | up | GRAP2 | protein_coding | 0.765996 | 0.660839 | 0.739854 | 0.028954 | 0.186922 | 0.115103 |
| ENSG00000100302 | 2.731568 | 0.000586 | 0.1623 | up | RASD2 | protein_coding | 0.178363 | 0.5278 | 0.471155 | 0.049553 | 0.058165 | 0.056284 |
| ENSG00000148346 | 3.210013 | 0.007066 | 0.784612 | up | LCN2 | protein_coding | 0.555143 | 0.22001 | 2.291308 | 0.064263 | 0.075431 | 0.145984 |
| ENSG00000090512 | 3.306394 | 0.01827 | 0.999963 | up | FETUB | protein_coding | 0.185541 | 0.235303 | 0.40843 | 0.034365 | 0 | 0.039033 |
| ENSG00000124440 | 1.581278 | 8.08E-05 | 0.035054 | up | HIF3A | protein_coding | 17.17989 | 18.24417 | 36.44438 | 4.555306 | 13.59918 | 4.999037 |
| ENSG00000197479 | -1.02151 | 0.045592 | 0.999963 | down | PCDHB11 | protein_coding | 0.130471 | 0.214489 | 0.319117 | 0.362478 | 0.583056 | 0.320222 |
| ENSG00000065618 | 4.659862 | 0.012751 | 1 | up | COL17A1 | protein_coding | 0.03184 | 0.022433 | 0.116816 | 0 | 0 | 0 |
| ENSG00000256371 | -4.80514 | 0.036867 | 1 | down | LRRC34P1 | processed_  pseudogene | 0 | 0 | 0 | 0.123188 | 0 | 0.769566 |
| ENSG00000148677 | 1.164107 | 0.001687 | 0.335684 | up | ANKRD1 | protein_coding | 56.24484 | 107.5403 | 103.537 | 22.81117 | 42.11881 | 44.63831 |
| ENSG00000134121 | 1.282726 | 0.016918 | 0.999963 | up | CHL1 | protein_coding | 0.29583 | 0.224461 | 0.651208 | 0.203715 | 0.156664 | 0.079788 |
| ENSG00000100739 | 2.508516 | 0.013621 | 0.999963 | up | BDKRB1 | protein_coding | 0.216447 | 0.457495 | 0.436757 | 0.066815 | 0.078427 | 0.037945 |
| ENSG00000041982 | 1.854341 | 3.06E-13 | 5.84E-09 | up | TNC | protein_coding | 7.534522 | 6.582288 | 9.783085 | 1.689064 | 2.934951 | 1.692293 |
| ENSG00000256574 | -1.85621 | 0.008847 | 0.884423 | down | OR13A1 | protein_coding | 0.283718 | 0.149921 | 0.052046 | 0.459803 | 0.719616 | 0.571997 |
| ENSG00000207563 | 1.360651 | 0.034958 | 0.999963 | up | MIR23B | miRNA | 20.07673 | 18.5655 | 29.69331 | 6.39119 | 18.4138 | 1.979812 |
| ENSG00000109265 | -1.02348 | 0.046545 | 0.999963 | down | KIAA1211 | protein_coding | 0.170948 | 0.138972 | 0.26052 | 0.414044 | 0.443118 | 0.235142 |
| ENSG00000148848 | -2.17684 | 0.00554 | 0.724698 | down | ADAM12 | protein_coding | 0.053666 | 0.016205 | 0.067506 | 0.092298 | 0.416687 | 0.112899 |
| ENSG00000173890 | -1.62347 | 0.008135 | 0.853353 | down | GPR160 | protein_coding | 0.388008 | 0.378516 | 0.229955 | 1.299096 | 0.583992 | 0.973238 |
| ENSG00000100867 | -4.34 | 0.01196 | 0.999963 | down | DHRS2 | protein_coding | 0 | 0 | 0.012595 | 0.010598 | 0.099516 | 0.132409 |
| ENSG00000266066 | -2.19418 | 0.049524 | 0.999963 | down | POLRMTP1 | processed_  pseudogene | 0.016591 | 0.035069 | 0.054784 | 0.03073 | 0.252493 | 0.17452 |
| ENSG00000100842 | 1.456182 | 0.000216 | 0.079224 | up | EFS | protein_coding | 1.171089 | 1.629564 | 1.976394 | 0.68686 | 0.615281 | 0.328487 |
| ENSG00000256609 | 3.549928 | 0.041489 | 1 | up | AC084880.3 | antisense_RNA | 0.674946 | 0.237768 | 0.619063 | 0 | 0 | 0.118325 |
| ENSG00000158246 | 1.815237 | 4.26E-07 | 0.000626 | up | FAM46B | protein_coding | 15.3943 | 17.73584 | 12.33294 | 2.665613 | 5.19616 | 4.541529 |
| ENSG00000207688 | -1.80866 | 0.014568 | 0.999963 | down | MIR548AA2 | miRNA | 7.528774 | 7.293589 | 2.071626 | 23.24069 | 8.865902 | 23.09781 |
| ENSG00000197921 | -1.44959 | 0.00477 | 0.654731 | down | HES5 | protein_coding | 0.652378 | 0.738701 | 1.23092 | 1.510378 | 1.97548 | 2.940916 |
| ENSG00000158445 | 1.207762 | 0.027284 | 0.999963 | up | KCNB1 | protein_coding | 0.563544 | 0.249057 | 0.941669 | 0.11861 | 0.434376 | 0.177831 |
| ENSG00000110436 | -2.38492 | 0.004374 | 0.623329 | down | SLC1A2 | protein_coding | 0.286181 | 0.225459 | 0.26344 | 0.115647 | 3.812184 | 0.459744 |
| ENSG00000242265 | -1.34257 | 0.022454 | 0.999963 | down | PEG10 | protein_coding | 0.091958 | 0.05831 | 0.16194 | 0.281027 | 0.189923 | 0.241818 |
| ENSG00000232876 | -1.49861 | 0.028705 | 0.999963 | down | AL353596.1 | antisense_RNA | 0.539244 | 0.732716 | 0.678305 | 1.569482 | 0.921121 | 2.430909 |
| ENSG00000232874 | -1.75228 | 0.014579 | 0.999963 | down | AC080129.2 | antisense_RNA | 0.283059 | 1.047008 | 1.090414 | 2.097067 | 2.461517 | 2.828522 |
| ENSG00000134594 | -1.35679 | 0.036341 | 0.999963 | down | RAB33A | protein_coding | 1.209412 | 1.227018 | 1.064906 | 4.614422 | 0.841377 | 2.646045 |
| ENSG00000183578 | -3.53173 | 0.021782 | 0.999963 | down | TNFAIP8L3 | protein_coding | 0 | 0.056867 | 0 | 0.373731 | 0.204718 | 0.056599 |
| ENSG00000217416 | -1.26779 | 0.020669 | 0.999963 | down | ISCA1P1 | processed_  pseudogene | 2.673331 | 4.653368 | 3.28855 | 10.63096 | 4.273467 | 8.270534 |
| ENSG00000134532 | 1.228008 | 0.023234 | 0.999963 | up | SOX5 | protein_coding | 0.593307 | 0.313513 | 0.768259 | 0.218163 | 0.123296 | 0.293684 |
| ENSG00000134548 | -1.19761 | 0.012851 | 0.999963 | down | SPX | protein_coding | 1.180138 | 1.445626 | 1.889328 | 3.005465 | 1.72016 | 4.372922 |
| ENSG00000099769 | -1.74043 | 0.019137 | 0.999963 | down | IGFALS | protein_coding | 0.170948 | 0.150553 | 0.094077 | 0.316622 | 0.464559 | 0.539443 |
| ENSG00000099860 | 1.073298 | 0.000463 | 0.134065 | up | GADD45B | protein_coding | 59.52339 | 68.48217 | 52.16431 | 22.71757 | 27.68747 | 30.14109 |
| ENSG00000144119 | -1.99543 | 1.44E-06 | 0.001621 | down | C1QL2 | protein_coding | 1.817089 | 2.0148 | 1.90161 | 3.668969 | 9.325571 | 8.616657 |
| ENSG00000099937 | -1.1987 | 0.005024 | 0.675746 | down | SERPIND1 | protein_coding | 3.496127 | 5.410267 | 3.243309 | 11.28561 | 5.619092 | 8.825884 |
| ENSG00000109906 | 2.140321 | 0.015742 | 0.999963 | up | ZBTB16 | protein_coding | 0.439901 | 0.523014 | 0.847306 | 0.012731 | 0.388522 | 0.02892 |
| ENSG00000183801 | 1.17665 | 0.032214 | 0.999963 | up | OLFML1 | protein_coding | 0.632117 | 0.552861 | 0.479818 | 0.262415 | 0.260633 | 0.183421 |
| ENSG00000252010 | 2.132286 | 0.039851 | 0.999963 | up | SCARNA5 | scaRNA | 9.481436 | 10.9524 | 0.728071 | 1.429387 | 0.239686 | 2.783214 |
| ENSG00000182601 | -2.45814 | 0.02228 | 0.999963 | down | HS3ST4 | protein_coding | 0.057001 | 0.04016 | 0.146387 | 0.985353 | 0.144575 | 0.059957 |
| ENSG00000216621 | -1.18325 | 0.011458 | 0.995415 | down | AL583835.2 | processed_  pseudogene | 0.661496 | 0.815604 | 1.092107 | 1.888832 | 2.277015 | 1.333624 |
| ENSG00000182752 | -1.92068 | 0.026206 | 0.999963 | down | PAPPA | protein_coding | 0.027766 | 0.005869 | 0.042785 | 0.138852 | 0.096583 | 0.035047 |
| ENSG00000108950 | -3.06451 | 0.036797 | 1 | down | FAM20A | protein_coding | 0 | 0.017534 | 0.018261 | 0.107555 | 0.072141 | 0.08726 |
| ENSG00000265683 | -2.53384 | 0.044701 | 0.999963 | down | SYPL1P2 | processed_  pseudogene | 0 | 0 | 0.460678 | 0.620178 | 0.727959 | 0.880522 |
| ENSG00000167244 | 2.688242 | 9.40E-06 | 0.006829 | up | IGF2 | protein_coding | 0.348692 | 2.084375 | 1.451189 | 0.201822 | 0.177672 | 0.171926 |
| ENSG00000226051 | -1.44582 | 0.031235 | 0.999963 | down | ZNF503-AS1 | lincRNA | 0.327191 | 0.380365 | 0.180061 | 0.84841 | 0.462362 | 0.929234 |
| ENSG00000157927 | 1.8889 | 0.039663 | 0.999963 | up | RADIL | protein_coding | 0.053676 | 0.147488 | 0.153603 | 0.019883 | 0.035008 | 0.033876 |
| ENSG00000265763 | -1.33244 | 0.018914 | 0.999963 | down | ZNF488 | protein_coding | 0.348156 | 0.331147 | 0.478994 | 0.338539 | 1.135354 | 1.226812 |
| ENSG00000035664 | -1.04717 | 0.04885 | 0.999963 | down | DAPK2 | protein_coding | 0.090315 | 0.103402 | 0.082838 | 0.188188 | 0.237255 | 0.126666 |
| ENSG00000118523 | 1.422663 | 7.66E-05 | 0.034023 | up | CTGF | protein_coding | 269.7612 | 433.9075 | 366.7289 | 72.67112 | 206.3507 | 109.5544 |
| ENSG00000060709 | -1.67779 | 0.023322 | 0.999963 | down | RIMBP2 | protein_coding | 0.038511 | 0.050875 | 0.063581 | 0.213986 | 0.177916 | 0.07089 |
| ENSG00000167588 | -1.71361 | 2.31E-05 | 0.013118 | down | GPD1 | protein_coding | 0.414669 | 1.064285 | 0.825872 | 2.742959 | 2.575728 | 1.765472 |
| ENSG00000153002 | -1.98867 | 0.002756 | 0.478459 | down | CPB1 | protein_coding | 0.170279 | 0.341916 | 0.2249 | 1.561138 | 0.351682 | 0.734351 |
| ENSG00000128016 | 1.62368 | 1.14E-08 | 3.10E-05 | up | ZFP36 | protein_coding | 16.32183 | 15.52087 | 28.46572 | 5.364487 | 8.408058 | 4.659469 |
| ENSG00000167600 | 1.667838 | 0.005187 | 0.692721 | up | CYP2S1 | protein_coding | 0.43751 | 0.670442 | 0.529697 | 0.12155 | 0.23779 | 0.13806 |
| ENSG00000167633 | -1.27039 | 0.023122 | 0.999963 | down | KIR3DL1 | protein_coding | 0.357794 | 0.756257 | 0.429605 | 0.843422 | 1.096072 | 1.505405 |
| ENSG00000143768 | -4.08572 | 0.026633 | 1 | down | LEFTY2 | protein_coding | 0 | 0.028123 | 0 | 0.024643 | 0.462813 | 0.055981 |
| ENSG00000104237 | 1.540279 | 2.01E-05 | 0.012365 | up | RP1 | protein_coding | 3.814314 | 3.686858 | 6.169944 | 0.873163 | 1.649173 | 1.776161 |
| ENSG00000177182 | -1.63732 | 3.48E-05 | 0.018482 | down | CLVS1 | protein_coding | 0.588077 | 0.42617 | 0.813703 | 2.007253 | 1.442879 | 1.732017 |
| ENSG00000153234 | -1.65104 | 0.016487 | 0.999963 | down | NR4A2 | protein_coding | 0.175281 | 0.185243 | 0.366552 | 1.331052 | 0.34296 | 0.368744 |
| ENSG00000167941 | -2.42034 | 0.003298 | 0.524845 | down | SOST | protein_coding | 0.02646 | 0.139818 | 0.232983 | 0.784121 | 0.287623 | 0.751468 |
| ENSG00000177283 | -1.12422 | 0.033224 | 0.999963 | down | FZD8 | protein_coding | 0.211416 | 0.462821 | 0.548493 | 0.629315 | 1.543029 | 0.428877 |
| ENSG00000261222 | 1.512191 | 0.036077 | 0.999963 | up | AC064805.1 | lincRNA | 0.7666 | 0.552386 | 0.479406 | 0.161347 | 0.056816 | 0.348201 |
| ENSG00000128383 | 4.484999 | 0.025915 | 0.999963 | up | APOBEC3A | protein_coding | 0.082351 | 0 | 1.132993 | 0 | 0.044759 | 0 |
| ENSG00000236194 | 2.79871 | 0.027752 | 0.999963 | up | AC099811.1 | sense_intronic | 0.284761 | 0.214961 | 0.40297 | 0.075346 | 0.04422 | 0 |
| ENSG00000069696 | -1.2225 | 0.018472 | 0.999963 | down | DRD4 | protein_coding | 0.796681 | 2.151669 | 0.974292 | 3.729919 | 2.357462 | 2.467446 |
| ENSG00000226942 | 3.562794 | 0.038632 | 1 | up | IL9RP3 | unprocessed_  pseudogene | 0.218128 | 0.115262 | 0.160054 | 0 | 0.039518 | 0 |
| ENSG00000080031 | 1.604917 | 0.002018 | 0.381304 | up | PTPRH | protein_coding | 0.690672 | 0.895815 | 0.639246 | 0.319807 | 0.290071 | 0.099067 |
| ENSG00000153551 | -2.8102 | 0.009689 | 0.928493 | down | CMTM7 | protein_coding | 0.058913 | 0.342439 | 0.162107 | 3.137099 | 0.12808 | 0.309845 |
| ENSG00000261251 | 1.02733 | 0.045042 | 0.999963 | up | Z97055.2 | antisense_RNA | 0.873554 | 1.099046 | 2.060298 | 0.654885 | 0.678263 | 0.481308 |
| ENSG00000138031 | 1.021293 | 0.014962 | 0.999963 | up | ADCY3 | protein_coding | 0.626652 | 0.80236 | 0.981527 | 0.212043 | 0.550186 | 0.367605 |
| ENSG00000114019 | 1.350512 | 0.006553 | 0.761718 | up | AMOTL2 | protein_coding | 40.65249 | 55.76631 | 71.14584 | 8.215078 | 45.27277 | 12.44597 |
| ENSG00000261399 | -1.46109 | 0.006681 | 0.761718 | down | AL031710.1 | antisense_RNA | 21.71628 | 18.55275 | 10.87751 | 47.44726 | 20.35485 | 61.00473 |
| ENSG00000138166 | 1.968143 | 0.000236 | 0.084972 | up | DUSP5 | protein_coding | 2.046833 | 2.993206 | 1.205005 | 0.749392 | 0.465686 | 0.325452 |
| ENSG00000187094 | -2.28697 | 0.000167 | 0.066435 | down | CCK | protein_coding | 2.391261 | 3.201068 | 0.833444 | 11.95823 | 4.722139 | 12.45065 |
| ENSG00000261634 | -1.25658 | 0.026519 | 0.999963 | down | AC026992.2 | antisense_RNA | 0.226423 | 0.239291 | 0.304592 | 0.465967 | 0.574295 | 0.661574 |
| ENSG00000163132 | -1.49535 | 0.006267 | 0.758658 | down | MSX1 | protein_coding | 6.528303 | 6.733465 | 2.798137 | 25.08382 | 8.80224 | 9.541014 |
| ENSG00000153898 | 1.583725 | 0.023945 | 0.999963 | up | MCOLN2 | protein_coding | 0.487539 | 0.246422 | 0.769915 | 0.098152 | 0.299545 | 0.089187 |
| ENSG00000114279 | -1.65014 | 0.027112 | 0.999963 | down | FGF12 | protein_coding | 0.067544 | 0.035692 | 0.049562 | 0.166803 | 0.159081 | 0.130254 |
| ENSG00000246145 | 1.090412 | 0.031513 | 0.999963 | up | RRS1-AS1 | lincRNA | 0.473925 | 0.368279 | 0.475598 | 0.129085 | 0.257583 | 0.205267 |
| ENSG00000138378 | -1.1967 | 0.010002 | 0.931764 | down | STAT4 | protein_coding | 0.539135 | 0.569775 | 1.139321 | 2.03706 | 1.078331 | 1.474453 |
| ENSG00000187242 | 5.187832 | 0.013172 | 0.999963 | up | KRT12 | protein_coding | 0.162982 | 0 | 0.609911 | 0 | 0 | 0 |
| ENSG00000246250 | -2.85988 | 0.043991 | 0.999963 | down | AC087521.2 | antisense_RNA | 0 | 0.029368 | 0.061171 | 0.128673 | 0.060414 | 0.350761 |
| ENSG00000163395 | 2.116924 | 0.001715 | 0.337592 | up | IGFN1 | protein_coding | 0.48954 | 0.457456 | 1.474638 | 0.062037 | 0.425711 | 0.065044 |
| ENSG00000284649 | -4.03731 | 0.046639 | 1 | down | AC009093.8 | unprocessed_  pseudogene | 0 | 0 | 0 | 0.249743 | 0.390861 | 0.189111 |
| ENSG00000137285 | 1.468623 | 6.10E-09 | 2.20E-05 | up | TUBB2B | protein_coding | 10.60489 | 11.93989 | 11.17482 | 3.459641 | 4.879622 | 3.327457 |
| ENSG00000113303 | 4.802149 | 0.001196 | 0.265491 | up | BTNL8 | protein_coding | 0.163816 | 0.40396 | 0.330556 | 0 | 0.029678 | 0 |
| ENSG00000186340 | -4.36482 | 0.036158 | 1 | down | THBS2 | protein_coding | 0 | 0 | 0 | 0.019397 | 0.011384 | 0.077112 |
| ENSG00000113368 | -1.15003 | 0.046311 | 0.999963 | down | LMNB1 | protein_coding | 0.227669 | 0.166574 | 0.154204 | 0.470331 | 0.399775 | 0.29474 |
| ENSG00000113396 | 1.456812 | 0.027558 | 0.999963 | up | SLC27A6 | protein_coding | 0.794041 | 0.339663 | 1.227702 | 0.420195 | 0.123305 | 0.218749 |
| ENSG00000137331 | 1.030068 | 0.014187 | 0.999963 | up | IER3 | protein_coding | 79.56566 | 110.2904 | 48.02899 | 40.24427 | 29.98948 | 39.73605 |
| ENSG00000260876 | -1.06203 | 0.018881 | 0.999963 | down | LINC01229 | lincRNA | 1.099836 | 0.947093 | 1.300197 | 1.395764 | 2.391082 | 2.699383 |
| ENSG00000259828 | 2.417718 | 4.18E-08 | 9.97E-05 | up | AL355596.1 | lincRNA | 1.568652 | 1.030089 | 2.631698 | 0.22566 | 0.397317 | 0.288351 |
| ENSG00000260830 | 4.670973 | 0.020501 | 1 | up | AL135744.1 | antisense_RNA | 1.064282 | 0.102252 | 0.212981 | 0 | 0 | 0 |
| ENSG00000113494 | 1.161442 | 0.019894 | 0.999963 | up | PRLR | protein_coding | 0.109344 | 0.192596 | 0.246428 | 0.067507 | 0.101879 | 0.060246 |
| ENSG00000162551 | 1.348451 | 0.001074 | 0.250134 | up | ALPL | protein_coding | 1.621929 | 1.763789 | 3.440974 | 0.718361 | 1.303135 | 0.519232 |
| ENSG00000245330 | 2.450773 | 0.032238 | 0.999963 | up | AP005717.1 | lincRNA | 0.320472 | 0.237079 | 0.176363 | 0.059356 | 0.034836 | 0.033709 |
| ENSG00000162598 | 2.093391 | 0.002211 | 0.409897 | up | C1orf87 | protein_coding | 0.646561 | 0.298946 | 0.644919 | 0.056134 | 0.175706 | 0.127518 |
| ENSG00000259969 | -1.63682 | 0.005671 | 0.732239 | down | AL049838.1 | sense_overlapping | 0.49629 | 0.524495 | 0.546239 | 1.321355 | 1.34869 | 1.827105 |
| ENSG00000235902 | -1.28178 | 0.027338 | 0.999963 | down | AC108472.1 | antisense_RNA | 0.787801 | 0.728501 | 1.192246 | 2.371077 | 2.354971 | 1.450154 |
| ENSG00000113578 | -1.38642 | 0.000904 | 0.224217 | down | FGF1 | protein_coding | 0.314626 | 0.601678 | 0.395761 | 0.832477 | 1.482016 | 0.977071 |
| ENSG00000137558 | 1.446745 | 6.23E-06 | 0.005662 | up | PI15 | protein_coding | 3.245377 | 3.124093 | 5.979876 | 1.021354 | 2.014471 | 1.217139 |
| ENSG00000172005 | -2.29167 | 0.012489 | 0.999963 | down | MAL | protein_coding | 0.109456 | 0.34703 | 0.180708 | 1.773879 | 0.356942 | 0.690798 |
| ENSG00000172020 | -4.495 | 0.014528 | 1 | down | GAP43 | protein_coding | 0 | 0 | 0 | 0.118587 | 0.139197 | 0.134695 |
| ENSG00000113763 | 1.768186 | 0.019512 | 0.999963 | up | UNC5A | protein_coding | 0.494907 | 0.421801 | 0.632574 | 0.088707 | 0.364433 | 0.016793 |
| ENSG00000162772 | 1.946057 | 0.013693 | 0.999963 | up | ATF3 | protein_coding | 2.199323 | 0.886909 | 0.509615 | 0.562783 | 0.28311 | 0.091318 |
| ENSG00000006016 | -3.57471 | 0.025305 | 0.999963 | down | CRLF1 | protein_coding | 0.067921 | 0 | 0 | 0.0629 | 0.701402 | 0.142888 |
| ENSG00000172201 | -3.44497 | 0.038525 | 0.999963 | down | ID4 | protein_coding | 0 | 0.027439 | 0.028576 | 0.456832 | 0.056445 | 0.02731 |
| ENSG00000186832 | 1.370417 | 0.045427 | 0.999963 | up | KRT16 | protein_coding | 0.444216 | 2.621159 | 1.874208 | 0.891317 | 0.563349 | 0.311503 |
| ENSG00000196159 | 1.018266 | 0.032205 | 0.999963 | up | FAT4 | protein_coding | 0.173631 | 0.143607 | 0.432065 | 0.090884 | 0.143606 | 0.103229 |
| ENSG00000147257 | 1.321379 | 0.04188 | 0.999963 | up | GPC3 | protein_coding | 1.232319 | 1.0519 | 0.469504 | 0.329198 | 0.206085 | 0.498551 |
| ENSG00000162946 | -1.47474 | 0.01272 | 0.999963 | down | DISC1 | protein_coding | 0.094834 | 0.045556 | 0.199268 | 0.359278 | 0.328002 | 0.181368 |
| ENSG00000123358 | 1.665662 | 0.001389 | 0.301114 | up | NR4A1 | protein_coding | 8.53038 | 4.89319 | 4.403651 | 3.076016 | 1.709563 | 0.741116 |
| ENSG00000137959 | -1.70415 | 0.027518 | 0.999963 | down | IFI44L | protein_coding | 0.041442 | 0.032848 | 0.31929 | 0.239865 | 0.540578 | 0.348731 |
| ENSG00000196353 | -2.044 | 9.59E-06 | 0.006829 | down | CPNE4 | protein_coding | 0.156878 | 0.130267 | 0.197334 | 0.601879 | 0.548131 | 0.683632 |
| ENSG00000231167 | -2.03359 | 0.032689 | 0.999963 | down | YBX1P2 | processed_  pseudogene | 0.060919 | 0.515045 | 0.134099 | 1.353962 | 0.463536 | 0.833014 |
| ENSG00000270876 | 1.472576 | 0.026357 | 0.999963 | up | ZNF30-AS1 | lincRNA | 1.655326 | 1.234871 | 2.464959 | 0.450869 | 0.740917 | 0.614534 |
| ENSG00000196569 | -1.89867 | 0.037053 | 0.999963 | down | LAMA2 | protein_coding | 0.031412 | 0.019918 | 0.020744 | 0.157085 | 0.07512 | 0.026433 |
| ENSG00000280392 | 4.622641 | 0.010099 | 1 | up | AC007496.3 | TEC | 0.106054 | 0.056041 | 0.097274 | 0 | 0 | 0 |
| ENSG00000231389 | 1.308512 | 0.040976 | 0.999963 | up | HLA-DPA1 | protein_coding | 0.399662 | 0.460773 | 0.159958 | 0.157019 | 0.144813 | 0.101913 |
| ENSG00000255320 | -2.08166 | 0.036014 | 0.999963 | down | AP000759.1 | antisense_RNA | 0.459881 | 0.486017 | 0.168722 | 2.129421 | 0.833165 | 1.451197 |
| ENSG00000147588 | -2.0727 | 0.010592 | 0.952617 | down | PMP2 | protein_coding | 0.084337 | 0.071304 | 0.11139 | 0.249928 | 0.788412 | 0.106453 |
| ENSG00000074181 | 1.370049 | 0.020621 | 0.999963 | up | NOTCH3 | protein_coding | 0.358151 | 0.615999 | 0.780665 | 0.065034 | 0.458019 | 0.147736 |
| ENSG00000108018 | -4.43935 | 0.013328 | 0.999963 | down | SORCS1 | protein_coding | 0 | 0 | 0.018422 | 0.286753 | 0.054582 | 0 |
| ENSG00000182022 | 1.275714 | 0.011017 | 0.974086 | up | CHST15 | protein_coding | 0.694433 | 0.747242 | 1.014466 | 0.198775 | 0.233321 | 0.478112 |
| ENSG00000157087 | -1.67424 | 0.004412 | 0.624179 | down | ATP2B2 | protein_coding | 0.066869 | 0.049469 | 0.110399 | 0.179585 | 0.407052 | 0.119573 |
| ENSG00000147689 | 1.078664 | 0.001099 | 0.252881 | up | FAM83A | protein_coding | 11.91649 | 14.38259 | 23.66063 | 7.680998 | 10.81906 | 4.059478 |
| ENSG00000279588 | 1.415166 | 0.007979 | 0.841905 | up | AC087762.2 | TEC | 1.032305 | 0.920507 | 2.254648 | 0.194186 | 0.859133 | 0.441124 |
| ENSG00000172716 | 3.368915 | 6.16E-06 | 0.005662 | up | SLFN11 | protein_coding | 0.217781 | 0.946202 | 0.466082 | 0.044818 | 0.052607 | 0.050906 |
| ENSG00000108255 | -3.63917 | 0.015606 | 0.999963 | down | CRYBA1 | protein_coding | 10.49269 | 17.57812 | 5.902679 | 373.925 | 6.927929 | 18.14954 |
| ENSG00000182256 | -1.03716 | 0.002511 | 0.44408 | down | GABRG3 | protein_coding | 0.412575 | 0.310591 | 0.559847 | 0.701343 | 0.810943 | 0.903614 |
| ENSG00000255690 | -1.66224 | 0.041265 | 0.999963 | down | TRIL | protein_coding | 0.061659 | 0.104261 | 0.013573 | 0.194143 | 0.241289 | 0.129714 |
| ENSG00000280649 | -1.01201 | 0.041625 | 0.999963 | down | AC245100.8 | TEC | 1.725641 | 0.972646 | 1.07628 | 2.503646 | 2.063382 | 2.6017 |
| ENSG00000172817 | 1.264033 | 0.040478 | 0.999963 | up | CYP7B1 | protein_coding | 0.497473 | 0.438121 | 0.912569 | 0.281537 | 0.180254 | 0.232567 |
| ENSG00000133328 | 1.167293 | 0.014158 | 0.999963 | up | HRASLS2 | protein_coding | 3.690824 | 3.727219 | 2.798463 | 1.063371 | 1.872262 | 1.466626 |
| ENSG00000123999 | -1.12306 | 7.02E-05 | 0.031935 | down | INHA | protein_coding | 7.088997 | 7.141376 | 7.39181 | 11.09513 | 18.6563 | 15.00054 |
| ENSG00000050767 | 1.318894 | 0.02157 | 0.999963 | up | COL23A1 | protein_coding | 3.002122 | 2.920598 | 5.120523 | 0.865357 | 3.241748 | 0.397342 |
| ENSG00000279970 | -2.16561 | 0.026137 | 0.999963 | down | AC023024.2 | TEC | 0 | 0.161355 | 0.100827 | 0.282783 | 0.365121 | 0.417552 |
| ENSG00000230847 | 1.903507 | 0.007797 | 0.834906 | up | AC044797.1 | unprocessed_  pseudogene | 0.896283 | 2.178605 | 2.071626 | 0.415012 | 0.487137 | 0.377107 |
| ENSG00000146966 | -1.90127 | 0.003293 | 0.524845 | down | DENND2A | protein_coding | 0.179233 | 0.154979 | 0.071735 | 0.648841 | 0.61991 | 0.239945 |
| ENSG00000278713 | -2.32177 | 0.046305 | 0.999963 | down | AC120114.3 | antisense_RNA | 0 | 0.488725 | 0.101797 | 0.685212 | 0.603221 | 1.361998 |
| ENSG00000156395 | -1.08594 | 0.001538 | 0.312412 | down | SORCS3 | protein_coding | 1.342525 | 1.485853 | 2.094296 | 2.036235 | 5.239863 | 2.757592 |
| ENSG00000264242 | 3.019299 | 0.041162 | 1 | up | AC011840.4 | unprocessed_  pseudogene | 0.485308 | 0.718044 | 0.427321 | 0.089886 | 0.105508 | 0 |
| ENSG00000181585 | -2.29021 | 0.008224 | 0.853597 | down | TMIE | protein_coding | 0.200189 | 0.141044 | 0.624289 | 3.120738 | 0.507755 | 0.491335 |
| ENSG00000254968 | -2.89298 | 0.034159 | 0.999963 | down | AP003063.1 | lincRNA | 0 | 0 | 0.235027 | 0.593249 | 0.541606 | 0.37435 |
| ENSG00000132692 | 1.526261 | 0.016214 | 0.999963 | up | BCAN | protein_coding | 0.280935 | 1.094823 | 0.657071 | 0.276427 | 0.286295 | 0.110815 |
| ENSG00000166025 | -1.2652 | 0.000385 | 0.122556 | down | AMOTL1 | protein_coding | 0.522412 | 0.322657 | 0.657131 | 0.823075 | 1.519239 | 1.070467 |
| ENSG00000166033 | -3.15528 | 0.016942 | 0.999963 | down | HTRA1 | protein_coding | 0.344477 | 0.879797 | 0.379147 | 10.68688 | 2.902007 | 0.181171 |
| ENSG00000142178 | -2.21389 | 0.000153 | 0.062244 | down | SIK1 | protein_coding | 0.439125 | 0.163793 | 0.497536 | 2.559583 | 0.786202 | 1.263433 |
| ENSG00000117228 | 1.744772 | 0.002259 | 0.414803 | up | GBP1 | protein_coding | 0.94244 | 3.093959 | 6.885853 | 0.761353 | 1.460385 | 0.738217 |
| ENSG00000166105 | 4.638997 | 0.012493 | 1 | up | GLB1L3 | protein_coding | 0.126064 | 0.033307 | 0.069376 | 0 | 0 | 0 |
| ENSG00000166145 | 1.758512 | 0.023435 | 0.999963 | up | SPINT1 | protein_coding | 0.57751 | 0.441963 | 0.54796 | 0.055326 | 0.064941 | 0.293258 |
| ENSG00000240596 | -2.84722 | 0.006349 | 0.758658 | down | KCNAB1-AS2 | antisense_RNA | 0.339355 | 0 | 0.249006 | 1.78085 | 1.598499 | 0.71391 |
| ENSG00000225156 | -2.06651 | 0.009838 | 0.928493 | down | AC012354.1 | lincRNA | 0.090663 | 0.119769 | 0.174629 | 0.944559 | 0.344933 | 0.190731 |
| ENSG00000240710 | 3.049181 | 0.026781 | 0.999963 | up | AL512306.3 | antisense_RNA | 0.356413 | 0.65917 | 1.372996 | 0 | 0.290571 | 0 |
| ENSG00000239881 | -1.4364 | 0.039643 | 0.999963 | down | RPS27P25 | processed_  pseudogene | 3.646426 | 2.126155 | 1.10715 | 8.034604 | 2.323565 | 7.00979 |
| ENSG00000215859 | -2.01866 | 0.041086 | 0.999963 | down | PDZK1P1 | unprocessed_  pseudogene | 0.052193 | 0.110319 | 0.172339 | 0.161117 | 1.19144 | 0.0549 |
| ENSG00000250358 | -1.96667 | 0.018523 | 0.999963 | down | LINC02200 | lincRNA | 0.296866 | 0.627475 | 0.435659 | 2.107723 | 0.645398 | 1.977666 |
| ENSG00000166482 | -3.0754 | 0.002034 | 0.381304 | down | MFAP4 | protein_coding | 0.629996 | 0.26632 | 0.312031 | 8.634662 | 0.890261 | 0.231935 |
| ENSG00000274414 | 1.419593 | 0.034381 | 0.999963 | up | AL121772.1 | lincRNA | 0.312758 | 0.605977 | 0.602413 | 0.096546 | 0.283312 | 0.16449 |
| ENSG00000127129 | 2.840878 | 3.39E-11 | 2.16E-07 | up | EDN2 | protein_coding | 8.042421 | 7.100176 | 7.394531 | 0.726623 | 0.799596 | 1.392729 |
| ENSG00000250644 | -1.9133 | 0.030558 | 0.999963 | down | AC068580.4 | protein_coding | 0.030142 | 0.031855 | 0.08294 | 0.111657 | 0.278506 | 0.126823 |
| ENSG00000127252 | -1.26338 | 0.04573 | 0.999963 | down | HRASLS | protein_coding | 1.084704 | 0.965346 | 0.377013 | 2.643465 | 1.303207 | 1.621366 |
| ENSG00000142871 | 1.756036 | 2.34E-05 | 0.013118 | up | CYR61 | protein_coding | 137.8464 | 204.2952 | 99.53786 | 27.14568 | 71.93085 | 32.51013 |
| ENSG00000166922 | 1.171867 | 0.037684 | 0.999963 | up | SCG5 | protein_coding | 1.868004 | 1.350744 | 1.352637 | 0.68286 | 0.427485 | 0.775613 |
| ENSG00000127362 | 2.521137 | 0.038404 | 0.999963 | up | TAS2R3 | protein_coding | 0.497473 | 0.233665 | 0.608379 | 0.153566 | 0.060085 | 0 |
| ENSG00000259054 | -3.29828 | 0.037217 | 0.999963 | down | LINC02332 | lincRNA | 0.157662 | 0.166622 | 0 | 0.876042 | 0.171382 | 1.824231 |
| ENSG00000250889 | -2.1568 | 0.046088 | 0.999963 | down | LINC01336 | lincRNA | 0.227931 | 0.120442 | 0.250871 | 0.633244 | 0.743295 | 1.078886 |
| ENSG00000176349 | 2.912337 | 0.000295 | 0.098957 | up | AC104129.1 | antisense_RNA | 0.258044 | 0.473652 | 0.4036 | 0 | 0.073815 | 0.071428 |
| ENSG00000260362 | -2.9905 | 0.031243 | 0.999963 | down | AC007218.1 | lincRNA | 0 | 0.292346 | 0 | 1.195487 | 0.501161 | 0.484954 |
| ENSG00000127530 | 3.871254 | 1.48E-07 | 0.000236 | up | OR7C1 | protein_coding | 0.217016 | 0.1529 | 0.31279 | 0.004785 | 0.011233 | 0.027175 |
| ENSG00000029153 | -2.59225 | 0.030915 | 0.999963 | down | ARNTL2 | protein_coding | 0.026908 | 0.066354 | 0.039489 | 0.681122 | 0.04875 | 0.009435 |
| ENSG00000259420 | -1.88138 | 0.001258 | 0.276089 | down | AC046168.2 | lincRNA | 1.708744 | 2.292046 | 1.519043 | 6.512287 | 2.571833 | 9.055966 |
| ENSG00000259426 | 1.688129 | 0.022009 | 0.999963 | up | AC027237.3 | antisense_RNA | 0.342281 | 1.374587 | 2.109687 | 0.126791 | 0.520892 | 0.43204 |
| ENSG00000259417 | -1.86522 | 0.003084 | 0.512157 | down | CTXND1 | protein_coding | 0.821445 | 1.754927 | 0.554137 | 6.584722 | 1.4306 | 2.620019 |
| ENSG00000176788 | -1.52049 | 0.01272 | 0.999963 | down | BASP1 | protein_coding | 4.018286 | 7.191907 | 6.099052 | 31.9906 | 10.32104 | 4.260778 |
| ENSG00000019991 | -1.50559 | 0.0389 | 0.999963 | down | HGF | protein_coding | 0.162585 | 0.096651 | 0.134211 | 0.122335 | 0.861572 | 0.181706 |
| ENSG00000186081 | 3.18397 | 0.000279 | 0.095267 | up | KRT5 | protein_coding | 0.98584 | 0.711519 | 3.175784 | 0.089069 | 0.444333 | 0 |
| ENSG00000234362 | -1.92253 | 0.019129 | 0.999963 | down | LINC01914 | lincRNA | 0.316554 | 0.418181 | 0.174207 | 1.099324 | 0.774226 | 1.331889 |
| ENSG00000175536 | -1.09351 | 0.028667 | 0.999963 | down | LIPT2 | protein_coding | 1.016286 | 2.148085 | 1.250166 | 2.934194 | 2.339408 | 3.395631 |
| ENSG00000248964 | -1.69229 | 0.045659 | 0.999963 | down | AC131254.1 | lincRNA | 0.130386 | 0.103347 | 0.107631 | 0.482988 | 0.318896 | 0.240009 |
| ENSG00000273906 | -1.79435 | 0.0048 | 0.654731 | down | AC011297.1 | sense_intronic | 0.259582 | 0.243853 | 0.222217 | 0.373944 | 1.097329 | 0.940489 |
| ENSG00000224965 | -2.26547 | 0.027164 | 0.999963 | down | KCNC4-AS1 | antisense_RNA | 0.08136 | 0.257953 | 0.179098 | 0.753458 | 0.442201 | 1.026961 |
| ENSG00000175497 | -2.37788 | 0.027952 | 0.999963 | down | DPP10 | protein_coding | 0.029081 | 0.020489 | 0.010669 | 0.035909 | 0.231821 | 0.061179 |
| ENSG00000102554 | 1.274328 | 0.008444 | 0.865443 | up | KLF5 | protein_coding | 7.884522 | 6.402762 | 10.78183 | 1.090507 | 5.750845 | 3.17736 |
| ENSG00000175600 | -1.03184 | 0.042615 | 0.999963 | down | SUGCT | protein_coding | 21.27241 | 18.68884 | 14.37377 | 34.7745 | 15.36204 | 49.92697 |
| ENSG00000258479 | -1.81246 | 0.019486 | 0.999963 | down | LINC00640 | lincRNA | 0.343828 | 0.363368 | 0.283824 | 1.273642 | 1.121242 | 0.904152 |
| ENSG00000209482 | 2.619682 | 0.042032 | 0.999963 | up | SNORD83A | snoRNA | 4.484244 | 4.062076 | 4.230478 | 1.186498 | 0 | 0.673831 |
| ENSG00000234578 | -3.65755 | 0.035333 | 1 | down | AL355483.4 | antisense_RNA | 0.129484 | 0 | 0 | 0.239824 | 0.84451 | 0.680999 |
| ENSG00000234546 | -2.19353 | 0.009098 | 0.891052 | down | LINC01759 | lincRNA | 0.185353 | 0.195887 | 0.136005 | 0.686603 | 0.94025 | 0.649888 |
| ENSG00000175832 | -2.37244 | 0.016696 | 0.999963 | down | ETV4 | protein_coding | 0.175382 | 0.052957 | 0.193033 | 0.208822 | 1.988139 | 0.13177 |
| ENSG00000175899 | -2.00397 | 0.043134 | 0.999963 | down | A2M | protein_coding | 0.125635 | 0.079665 | 0.013828 | 0.093078 | 0.150224 | 0.568249 |
| ENSG00000234665 | 1.057295 | 0.003777 | 0.57707 | up | AL512625.3 | lincRNA | 4.748564 | 4.255629 | 3.554007 | 2.216352 | 1.899532 | 1.638309 |
| ENSG00000136231 | -1.08941 | 0.013261 | 0.999963 | down | IGF2BP3 | protein_coding | 0.286389 | 0.272398 | 0.441297 | 0.450869 | 0.887232 | 0.662732 |
| ENSG00000126890 | 4.478744 | 0.026764 | 0.999963 | up | CTAG2 | protein_coding | 0 | 1.424931 | 0.13491 | 0 | 0 | 0.064465 |
| ENSG00000185352 | -5.0751 | 0.027635 | 1 | down | HS6ST3 | protein_coding | 0 | 0 | 0 | 0.129992 | 0 | 0.008203 |
| ENSG00000234770 | 3.58361 | 0.044691 | 1 | up | GULOP | unitary_  pseudogene | 0.244081 | 0.687874 | 0.179098 | 0 | 0.08844 | 0 |
| ENSG00000112414 | -2.94459 | 0.048549 | 0.999963 | down | ADGRG6 | protein_coding | 0 | 0.009154 | 0.038134 | 0.008021 | 0.338958 | 0.018222 |
| ENSG00000185432 | 1.070053 | 2.70E-06 | 0.002867 | up | METTL7A | protein_coding | 72.42889 | 77.87057 | 112.3716 | 32.32742 | 53.02324 | 32.91786 |
| ENSG00000185499 | 1.36218 | 0.00044 | 0.12963 | up | MUC1 | protein_coding | 0.831866 | 0.95626 | 0.915589 | 0.351397 | 0.34901 | 0.29167 |
| ENSG00000087074 | 1.28614 | 0.000187 | 0.0713 | up | PPP1R15A | protein_coding | 9.827298 | 11.19718 | 12.81626 | 4.763706 | 6.676528 | 2.126619 |
| ENSG00000283913 | -1.72287 | 0.010579 | 0.952617 | down | AL512662.2 | processed_  transcript | 0.093007 | 0.294878 | 0.204735 | 0.545499 | 0.606602 | 0.652205 |
| ENSG00000244306 | 1.176404 | 0.014068 | 0.999963 | up | DUXAP10 | processed_  pseudogene | 1.538718 | 1.419449 | 0.645857 | 0.458888 | 0.595337 | 0.507503 |
| ENSG00000258955 | -2.07141 | 0.00024 | 0.085002 | down | LINC00519 | lincRNA | 1.160042 | 1.320274 | 1.178579 | 6.363076 | 2.521972 | 5.068552 |
| ENSG00000244405 | -1.03174 | 3.38E-05 | 0.018443 | down | ETV5 | protein_coding | 3.138363 | 3.801711 | 3.861559 | 7.402988 | 6.871186 | 6.33755 |
| ENSG00000122194 | -4.75409 | 0.033773 | 1 | down | PLG | protein_coding | 0 | 0 | 0 | 0.082245 | 0 | 0.210188 |
| ENSG00000171227 | -1.58666 | 0.039524 | 0.999963 | down | TMEM37 | protein_coding | 0.576849 | 0.266714 | 0.119045 | 1.535841 | 0.587855 | 0.682613 |
| ENSG00000205086 | -2.35845 | 0.040717 | 0.999963 | down | C2orf91 | protein_coding | 0.03271 | 0.017285 | 0.018001 | 0.136315 | 0.088892 | 0.103221 |
| ENSG00000268654 | -1.28398 | 0.037891 | 0.999963 | down | MIMT1 | lincRNA | 0.566117 | 0.498575 | 0.363471 | 1.441733 | 0.769224 | 1.042087 |
| ENSG00000254038 | -3.68846 | 0.031586 | 1 | down | AC092818.1 | lincRNA | 0 | 0 | 0.104824 | 0.529189 | 0.621157 | 0.200357 |
| ENSG00000087495 | -1.16228 | 0.006223 | 0.758658 | down | PHACTR3 | protein_coding | 1.093117 | 0.848747 | 1.350455 | 3.160879 | 1.600482 | 1.971103 |
| ENSG00000171346 | 2.086423 | 0.001114 | 0.25319 | up | KRT15 | protein_coding | 0.522931 | 0.466682 | 0.639513 | 0.06457 | 0.290534 | 0.03667 |
| ENSG00000171345 | 1.353856 | 9.65E-06 | 0.006829 | up | KRT19 | protein_coding | 346.8445 | 299.9726 | 265.0872 | 87.57894 | 131.5451 | 121.3041 |
| ENSG00000244734 | -2.25926 | 5.78E-06 | 0.005662 | down | HBB | protein_coding | 29.98131 | 22.79442 | 13.49502 | 68.70785 | 77.43824 | 149.3972 |
| ENSG00000048052 | -1.20354 | 0.014618 | 0.999963 | down | HDAC9 | protein_coding | 0.200664 | 0.079525 | 0.255369 | 0.278745 | 0.593028 | 0.310011 |
| ENSG00000268812 | -3.08759 | 0.042692 | 1 | down | AC004264.1 | antisense_RNA | 0.076647 | 0 | 0.084361 | 0.567846 | 0.166633 | 0.483732 |
| ENSG00000205279 | 1.253129 | 0.039288 | 0.999963 | up | CTXN3 | protein_coding | 1.943044 | 2.440916 | 1.775442 | 0.33951 | 1.912866 | 0.424189 |
| ENSG00000171401 | 5.974555 | 0.018417 | 0.999963 | up | KRT13 | protein_coding | 0.265319 | 0 | 7.008531 | 0.061426 | 0.036051 | 0 |
| ENSG00000254396 | 3.118452 | 0.041544 | 1 | up | AL355432.1 | lincRNA | 0.323711 | 0.342107 | 1.425161 | 0.099927 | 0.117293 | 0 |
| ENSG00000230387 | -3.27263 | 0.002704 | 0.473684 | down | AL118508.1 | lincRNA | 0.051904 | 0.109708 | 0.085692 | 1.874617 | 0.169262 | 0.13649 |
| ENSG00000205359 | -2.93359 | 0.018603 | 0.999963 | down | SLCO6A1 | protein_coding | 0.022632 | 0 | 0.04982 | 0.188631 | 0.17221 | 0.142835 |
| ENSG00000171522 | 1.291543 | 0.027062 | 0.999963 | up | PTGER4 | protein_coding | 0.806584 | 0.869138 | 0.713692 | 0.087877 | 0.498556 | 0.365984 |
| ENSG00000122679 | -5.32772 | 0.040175 | 0.999963 | down | RAMP3 | protein_coding | 0 | 0 | 0 | 0.97978 | 0 | 0 |
| ENSG00000278484 | -1.1679 | 0.007632 | 0.832853 | down | AC010998.3 | lincRNA | 3.472065 | 2.955895 | 3.396898 | 7.681214 | 4.822584 | 7.710077 |
| ENSG00000229425 | 1.083911 | 0.033953 | 0.999963 | up | AJ009632.2 | lincRNA | 0.915152 | 0.639574 | 1.28344 | 0.191371 | 0.770157 | 0.341573 |
| ENSG00000205670 | 1.578507 | 0.020513 | 0.999963 | up | SMIM11A | protein_coding | 3.801551 | 0.484882 | 4.54486 | 0.971178 | 0.783722 | 0.930736 |
| ENSG00000122877 | 3.94576 | 0.003251 | 0.524845 | up | EGR2 | protein_coding | 3.690824 | 5.460809 | 0.338524 | 0.075955 | 0.57951 | 0.043136 |
| ENSG00000122862 | -2.76312 | 0.017903 | 0.999963 | down | SRGN | protein_coding | 0 | 0.159726 | 0.055449 | 0.279927 | 0.547626 | 0.529917 |
| ENSG00000254686 | -1.6335 | 0.041754 | 0.999963 | down | AL138812.1 | lincRNA | 0.646047 | 0.819315 | 0.426641 | 1.675205 | 1.123622 | 2.582303 |
| ENSG00000171819 | 1.32099 | 4.49E-09 | 2.14E-05 | up | ANGPTL7 | protein_coding | 20.42183 | 19.22589 | 23.6145 | 6.900034 | 10.58216 | 6.721749 |
| ENSG00000254671 | -3.35547 | 0.016957 | 0.999963 | down | STT3A-AS1 | antisense_RNA | 0 | 0.149921 | 0.156137 | 0.788233 | 1.387831 | 0.746083 |
| ENSG00000131068 | -1.76555 | 0.016455 | 0.999963 | down | DEFB118 | protein_coding | 0.578095 | 0.277704 | 0.289217 | 1.89809 | 0.628399 | 1.105595 |
| ENSG00000179059 | 1.918463 | 0.00648 | 0.761718 | up | ZFP42 | protein_coding | 0.482086 | 0.4367 | 0.480071 | 0.042519 | 0.199633 | 0.120735 |
| ENSG00000277481 | -3.74633 | 0.027412 | 1 | down | PKD1L3 | protein_coding | 0 | 0 | 0.012884 | 0.075882 | 0.038173 | 0.049251 |
| ENSG00000169618 | -1.66522 | 0.030996 | 0.999963 | down | PROKR1 | protein_coding | 0.028485 | 0.060207 | 0.141082 | 0.158274 | 0.356079 | 0.164791 |
| ENSG00000253408 | -4.04351 | 0.046225 | 1 | down | AC083973.1 | antisense_RNA | 0 | 0 | 0 | 0.276607 | 0.243509 | 0.157089 |
| ENSG00000106236 | -3.4956 | 0.015653 | 0.999963 | down | NPTX2 | protein_coding | 0.06762 | 0.047642 | 0 | 0.229609 | 1.274063 | 0 |
| ENSG00000170820 | -1.90828 | 1.03E-07 | 0.000197 | down | FSHR | protein_coding | 1.114848 | 0.924083 | 0.842094 | 3.137785 | 3.374197 | 3.678961 |
| ENSG00000179388 | 3.026502 | 0.009093 | 0.891052 | up | EGR3 | protein_coding | 0.266673 | 0.281828 | 0.123584 | 0 | 0.091541 | 0 |
| ENSG00000155368 | -1.00267 | 0.030917 | 0.999963 | down | DBI | protein_coding | 211.556 | 199.1358 | 125.7713 | 404.0823 | 181.7251 | 404.7627 |
| ENSG00000106366 | -1.8962 | 0.019242 | 0.999963 | down | SERPINE1 | protein_coding | 0.763105 | 1.088738 | 0.734919 | 0.512352 | 9.186802 | 0.82275 |
| ENSG00000277734 | -1.76715 | 0.004796 | 0.654731 | down | TRAC | TR_C_gene | 1.06002 | 1.911035 | 1.098075 | 3.753395 | 1.83006 | 6.68998 |
| ENSG00000228973 | -3.58358 | 0.039157 | 1 | down | AC009955.1 | antisense_RNA | 0 | 0 | 0.247168 | 1.039828 | 1.464648 | 0.472428 |
| ENSG00000204929 | -1.42977 | 0.042556 | 0.999963 | down | AC007389.1 | processed_  transcript | 0.096142 | 0.457224 | 0.476179 | 0.979377 | 0.83606 | 0.707895 |
| ENSG00000096696 | 1.320474 | 0.000722 | 0.186295 | up | DSP | protein_coding | 1.267349 | 1.096448 | 1.880381 | 0.276155 | 0.736087 | 0.581588 |
| ENSG00000180483 | -2.16852 | 0.002036 | 0.381304 | down | DEFB119 | protein_coding | 3.28631 | 1.800854 | 1.071721 | 10.93358 | 2.910744 | 11.65054 |
| ENSG00000214491 | 1.606324 | 0.000658 | 0.172046 | up | SEC14L6 | protein_coding | 0.663836 | 1.251435 | 1.520536 | 0.465225 | 0.370552 | 0.207592 |
| ENSG00000131686 | -1.12757 | 0.001447 | 0.301114 | down | CA6 | protein_coding | 2.608765 | 2.800102 | 4.845357 | 4.567582 | 9.703661 | 6.688662 |
| ENSG00000180638 | 1.149496 | 0.036398 | 0.999963 | up | SLC47A2 | protein_coding | 0.429951 | 0.64027 | 0.451713 | 0.253379 | 0.233682 | 0.164455 |
| ENSG00000224184 | -1.61811 | 0.010135 | 0.935038 | down | MIR3681HG | lincRNA | 0.306658 | 0.206236 | 0.153419 | 0.748695 | 0.545469 | 0.645124 |
| ENSG00000263711 | -3.69603 | 0.027944 | 0.999963 | down | AC079062.1 | processed_  transcript | 0 | 0 | 0.037109 | 0.031224 | 0.40315 | 0.035465 |
| ENSG00000082438 | -1.28716 | 0.035028 | 0.999963 | down | COBLL1 | protein_coding | 0.038344 | 0.05403 | 0.182878 | 0.22489 | 0.229241 | 0.147885 |
| ENSG00000180914 | 1.016718 | 0.023648 | 0.999963 | up | OXTR | protein_coding | 1.826843 | 2.181209 | 6.047464 | 0.981498 | 2.031287 | 1.481532 |
| ENSG00000189221 | 1.084252 | 1.95E-05 | 0.012365 | up | MAOA | protein_coding | 2.730646 | 2.826696 | 3.239503 | 1.357666 | 1.33815 | 1.188931 |
| ENSG00000165409 | 1.404718 | 0.005941 | 0.746475 | up | TSHR | protein_coding | 0.373196 | 0.690209 | 0.572124 | 0.123431 | 0.304253 | 0.168236 |
| ENSG00000180999 | -1.06617 | 0.037182 | 0.999963 | down | C1orf105 | protein_coding | 30.89218 | 23.76761 | 11.96846 | 38.56421 | 29.61786 | 62.12926 |
| ENSG00000273132 | -1.49539 | 0.020047 | 0.999963 | down | AL355312.3 | antisense_RNA | 11.31327 | 4.328975 | 3.649692 | 21.49578 | 8.057129 | 21.33798 |
| ENSG00000263961 | 1.821452 | 1.30E-05 | 0.008874 | up | C1orf186 | protein_coding | 7.821937 | 5.13757 | 15.72984 | 1.895547 | 3.734779 | 1.960787 |
| ENSG00000018236 | 1.832309 | 0.015171 | 0.999963 | up | CNTN1 | protein_coding | 0.197127 | 0.104165 | 0.313397 | 0.030426 | 0.059522 | 0.069117 |
| ENSG00000273350 | 4.106288 | 0.039578 | 1 | up | AC004832.5 | sense_intronic | 0.303529 | 0.481169 | 0.835194 | 0 | 0 | 0 |
| ENSG00000224594 | 1.170976 | 0.049491 | 0.999963 | up | RPL29P19 | processed_  pseudogene | 2.381384 | 4.194535 | 2.766672 | 0.857632 | 1.725737 | 1.391607 |
| ENSG00000165694 | -1.50847 | 0.003669 | 0.565051 | down | FRMD7 | protein_coding | 0.152239 | 0.301671 | 0.230397 | 0.652055 | 0.599889 | 0.560472 |
| ENSG00000141744 | 2.484142 | 0.019671 | 0.999963 | up | PNMT | protein_coding | 0.388556 | 0.410638 | 0.641493 | 0.134937 | 0.105592 | 0 |
| ENSG00000116774 | -1.57041 | 0.002437 | 0.439089 | down | OLFML3 | protein_coding | 6.578402 | 6.640499 | 4.578063 | 19.86076 | 6.733973 | 21.2242 |
| ENSG00000116741 | -2.65844 | 7.86E-06 | 0.006258 | down | RGS2 | protein_coding | 2.337219 | 2.992556 | 1.681985 | 27.05549 | 6.05835 | 8.273588 |
| ENSG00000165810 | 1.821443 | 0.004738 | 0.654731 | up | BTNL9 | protein_coding | 1.095437 | 0.973931 | 1.952064 | 0.080512 | 0.81274 | 0.237766 |
| ENSG00000165973 | -1.6479 | 0.024997 | 0.999963 | down | NELL1 | protein_coding | 0.091405 | 0.11592 | 0.100605 | 0.474029 | 0.258334 | 0.173063 |
| ENSG00000248713 | -3.83359 | 0.027984 | 1 | down | AC083902.2 | protein_coding | 0 | 0 | 0.006641 | 0.011176 | 0.026236 | 0.050774 |
| ENSG00000272677 | -1.29837 | 0.041454 | 0.999963 | down | AC124016.1 | antisense_RNA | 2.261268 | 2.389778 | 0.973898 | 3.277726 | 2.778651 | 6.72198 |
| ENSG00000139890 | -1.57938 | 0.043389 | 0.999963 | down | REM2 | protein_coding | 0.253178 | 0.200675 | 0.069665 | 0.381 | 0.447214 | 0.665771 |
| ENSG00000101230 | 1.685551 | 0.008891 | 0.884423 | up | ISM1 | protein_coding | 1.642897 | 0.843328 | 2.169894 | 0.108675 | 0.918441 | 0.394995 |
| ENSG00000101306 | 1.484998 | 0.00282 | 0.484168 | up | MYLK2 | protein_coding | 0.802925 | 0.565704 | 0.906395 | 0.247856 | 0.358069 | 0.173245 |
| ENSG00000164949 | -1.3622 | 0.000951 | 0.226922 | down | GEM | protein_coding | 2.508171 | 2.03901 | 2.578586 | 8.167924 | 3.74509 | 4.928609 |
| ENSG00000149380 | -4.5814 | 0.01652 | 1 | down | P4HA3 | protein_coding | 0 | 0 | 0 | 0.042907 | 0.075546 | 0.170574 |
| ENSG00000125378 | 1.190777 | 0.001419 | 0.301114 | up | BMP4 | protein_coding | 59.80996 | 58.44487 | 56.11581 | 12.64175 | 31.92059 | 28.1501 |
| ENSG00000101384 | -2.02117 | 0.007869 | 0.834906 | down | JAG1 | protein_coding | 0.080499 | 0.414737 | 0.764186 | 1.42574 | 3.248598 | 0.285776 |
| ENSG00000125354 | -1.44207 | 0.038398 | 0.999963 | down | 6-Sep | protein_coding | 0.142615 | 0.15072 | 0.042809 | 0.420229 | 0.281863 | 0.190924 |
| ENSG00000101347 | 2.10619 | 0.000186 | 0.0713 | up | SAMHD1 | protein_coding | 2.111697 | 1.50573 | 3.864379 | 0.153149 | 1.037102 | 0.48171 |
| ENSG00000101342 | -1.2435 | 0.012081 | 0.999963 | down | TLDC2 | protein_coding | 1.227085 | 0.988055 | 0.932547 | 1.866898 | 1.619691 | 3.319013 |
| ENSG00000198342 | 1.193609 | 0.042068 | 0.999963 | up | ZNF442 | protein_coding | 0.313144 | 0.361966 | 0.16156 | 0.11781 | 0.148922 | 0.09264 |
| ENSG00000149452 | -1.00084 | 0.043743 | 0.999963 | down | SLC22A8 | protein_coding | 0.19274 | 0.186719 | 0.141425 | 0.327234 | 0.25316 | 0.388578 |
| ENSG00000149451 | 1.371611 | 0.01514 | 0.999963 | up | ADAM33 | protein_coding | 1.290969 | 2.431317 | 3.916577 | 0.536457 | 2.015003 | 0.365595 |
| ENSG00000125430 | -1.83661 | 0.01512 | 0.999963 | down | HS3ST3B1 | protein_coding | 0.079375 | 0.011984 | 0.149765 | 0.315029 | 0.345126 | 0.143128 |
| ENSG00000101542 | 1.324831 | 9.36E-07 | 0.001117 | up | CDH20 | protein_coding | 10.51918 | 7.654323 | 10.26652 | 3.164913 | 4.839652 | 2.935209 |
| ENSG00000272942 | -3.87875 | 0.019039 | 1 | down | AL022324.3 | lincRNA | 0 | 0 | 0.089669 | 0.377233 | 0.354234 | 0.514168 |
| ENSG00000198468 | -1.12907 | 0.006742 | 0.761718 | down | FLVCR1-AS1 | lincRNA | 1.711243 | 1.400947 | 1.193749 | 2.700752 | 2.410337 | 3.650695 |
| ENSG00000150551 | -2.26354 | 0.006376 | 0.758658 | down | LYPD1 | protein_coding | 1.319493 | 0.442153 | 0.566748 | 8.583447 | 1.189429 | 0.947853 |
| ENSG00000198650 | -1.46272 | 0.025863 | 0.999963 | down | TAT | protein_coding | 0.091676 | 0.20992 | 0.084086 | 0.339595 | 0.282351 | 0.369651 |
| ENSG00000233435 | 1.257905 | 0.046693 | 0.999963 | up | AGGF1P2 | processed_  pseudogene | 0.606842 | 0.702409 | 1.558474 | 0.133805 | 0.628236 | 0.364752 |
| ENSG00000125740 | 3.241152 | 6.42E-05 | 0.030668 | up | FOSB | protein_coding | 0.564243 | 0.528159 | 1.011393 | 0.164224 | 0.035048 | 0 |
| ENSG00000198719 | -1.1928 | 0.043051 | 0.999963 | down | DLL1 | protein_coding | 0.460171 | 0.628167 | 1.181797 | 1.384996 | 3.272232 | 0.463869 |
| ENSG00000198846 | -3.40531 | 0.019319 | 0.999963 | down | TOX | protein_coding | 0 | 0 | 0.048644 | 0.300143 | 0.112097 | 0.030992 |
| ENSG00000243089 | 1.329806 | 0.016124 | 0.999963 | up | AC107029.1 | lincRNA | 4.973308 | 4.011118 | 9.363156 | 0.969612 | 3.556627 | 2.340294 |
| ENSG00000184254 | 1.165984 | 0.001935 | 0.373296 | up | ALDH1A3 | protein_coding | 24.58188 | 16.3413 | 24.88802 | 5.515222 | 16.20114 | 7.226853 |
| ENSG00000159251 | 4.889865 | 0.000733 | 0.186723 | up | ACTC1 | protein_coding | 0.177859 | 0.125312 | 0.212073 | 0 | 0.016111 | 0 |
| ENSG00000159248 | 1.947367 | 0.001552 | 0.312412 | up | GJD2 | protein_coding | 0.631958 | 0.378462 | 0.788303 | 0.117048 | 0.13739 | 0.177263 |
| ENSG00000111319 | 1.089353 | 4.67E-07 | 0.000636 | up | SCNN1A | protein_coding | 38.98662 | 47.33643 | 47.62478 | 17.29131 | 26.4157 | 16.55057 |
| ENSG00000267194 | -1.01076 | 0.024068 | 0.999963 | down | AC002546.1 | lincRNA | 0.364562 | 0.770561 | 0.535005 | 1.012836 | 1.215276 | 0.945893 |
| ENSG00000257773 | -2.12195 | 0.043178 | 0.999963 | down | ST13P3 | processed_  pseudogene | 0.219307 | 0.057943 | 0.120689 | 0.253868 | 0.417183 | 0.922723 |
| ENSG00000135362 | 1.088446 | 0.002461 | 0.439249 | up | PRR5L | protein_coding | 3.56164 | 2.94578 | 4.073495 | 1.032525 | 1.632791 | 1.938335 |
| ENSG00000135373 | 2.656766 | 0.009455 | 0.92132 | up | EHF | protein_coding | 0.289798 | 0.142925 | 0.786779 | 0 | 0.147007 | 0.040644 |
| ENSG00000184557 | 1.663634 | 0.000432 | 0.12963 | up | SOCS3 | protein_coding | 4.051237 | 7.739586 | 17.12539 | 2.535522 | 4.427962 | 1.545325 |
| ENSG00000267394 | -4.08609 | 0.043416 | 1 | down | AC004596.1 | antisense_RNA | 0 | 0 | 0 | 0.42696 | 0.250581 | 0.363716 |
| ENSG00000184613 | -1.1757 | 0.020299 | 0.999963 | down | NELL2 | protein_coding | 0.320126 | 0.445156 | 0.593423 | 0.452492 | 1.648337 | 0.850683 |
| ENSG00000160678 | 1.325693 | 0.0058 | 0.738485 | up | S100A1 | protein_coding | 1.091304 | 1.276018 | 0.817796 | 0.365547 | 0.403835 | 0.439623 |
| ENSG00000267498 | -1.02586 | 0.029529 | 0.999963 | down | AC007786.1 | lincRNA | 4.549298 | 3.809986 | 3.21214 | 6.51821 | 5.225082 | 9.931639 |
| ENSG00000267414 | -1.06924 | 0.011858 | 0.999963 | down | AC120049.1 | lincRNA | 1.620904 | 1.027813 | 1.102861 | 2.729234 | 2.050271 | 2.603956 |
| ENSG00000169245 | -4.52388 | 0.020725 | 1 | down | CXCL10 | protein_coding | 0 | 0 | 0 | 0.383392 | 0.225011 | 0.054434 |
| ENSG00000169282 | -1.32601 | 0.023116 | 0.999963 | down | KCNAB1 | protein_coding | 0.137159 | 0.101468 | 0.226445 | 0.292145 | 0.313099 | 0.447246 |
| ENSG00000121207 | 1.490247 | 0.032513 | 0.999963 | up | LRAT | protein_coding | 0.1095 | 0.27002 | 0.455299 | 0.067603 | 0.145479 | 0.063988 |
| ENSG00000160868 | -4.65494 | 0.009869 | 0.928493 | down | CYP3A4 | protein_coding | 0 | 0.027462 | 0 | 0.072193 | 0.028246 | 0.519327 |
| ENSG00000169218 | -2.69804 | 0.049574 | 0.999963 | down | RSPO1 | protein_coding | 0.092877 | 0.024539 | 0.127781 | 1.376175 | 0.02524 | 0.024423 |
| ENSG00000170345 | 2.495165 | 6.46E-07 | 0.000823 | up | FOS | protein_coding | 62.29584 | 29.86322 | 17.59922 | 7.625919 | 6.958662 | 4.864821 |
| ENSG00000277196 | 1.138738 | 0.00494 | 0.669153 | up | AC007325.2 | protein_coding | 9.286792 | 4.573002 | 9.218808 | 2.976113 | 5.446298 | 1.916425 |
| ENSG00000170477 | 1.086262 | 0.003558 | 0.556978 | up | KRT4 | protein_coding | 56.43975 | 31.20933 | 33.75131 | 14.33033 | 27.70939 | 14.86038 |
| ENSG00000072041 | 1.00121 | 0.009796 | 0.928493 | up | SLC6A15 | protein_coding | 2.681775 | 1.99052 | 3.253714 | 1.709589 | 1.450789 | 0.616654 |
| ENSG00000228208 | -2.60813 | 0.004284 | 0.623329 | down | C1orf143 | lincRNA | 0.224567 | 0.158219 | 0.164779 | 1.386437 | 0.569585 | 1.102331 |
| ENSG00000096060 | 2.118042 | 3.25E-11 | 2.16E-07 | up | FKBP5 | protein_coding | 15.54407 | 11.55845 | 18.97719 | 2.137111 | 4.784107 | 3.190293 |
| ENSG00000228223 | 1.004095 | 0.039207 | 0.999963 | up | HCG11 | lincRNA | 0.406002 | 0.270995 | 0.56446 | 0.1781 | 0.29035 | 0.123622 |
| ENSG00000170579 | -1.12997 | 0.031925 | 0.999963 | down | DLGAP1 | protein_coding | 0.10017 | 0.144358 | 0.120274 | 0.303593 | 0.306861 | 0.153258 |
| ENSG00000145569 | 1.644778 | 0.046381 | 0.999963 | up | FAM105A | protein_coding | 0.439894 | 0.268208 | 0.391058 | 0.015668 | 0.275869 | 0.071186 |
| ENSG00000145536 | -5.61236 | 0.000945 | 0.226922 | down | ADAMTS16 | protein_coding | 0 | 0 | 0 | 0.045277 | 0.279016 | 0.03857 |
| ENSG00000253301 | 3.826072 | 0.021538 | 1 | up | LINC01606 | lincRNA | 0.132761 | 0.084184 | 0.233796 | 0 | 0.028863 | 0 |
